# The extra-embryonic space and the local contour are crucial geometric constraints regulating cell arrangement

**DOI:** 10.1242/dev.200401

**Published:** 2022-05-12

**Authors:** Sungrim Seirin-Lee, Kazunori Yamamoto, Akatsuki Kimura

**Affiliations:** 1 Institute for the Advanced Study of Human Biology (ASHBi), Kyoto University Institute for Advanced Study, Kyoto University, Kyoto 606-8315, Japan; 2 JST CREST, 4-1-8 Honcho, Kawaguchi, Saitama 332-0012, Japan; 3 Department of Applied Bioscience, Faculty of Applied Bioscience, Kanagawa Institute of Technology, Atsugi 243-0292, Japan; 4 Cell Architecture Laboratory, Department of Chromosome Science, National Institute of Genetics, Mishima 411-8540, Japan; 5 Department of Genetics, The Graduate University for Advanced Studies, Sokendai, Mishima 411-8540, Japan

**Keywords:** Cell arrangement, Extra-embryonic space, Geometric constraint, Phase-field modeling, Embryogenesis, *Caenorhabditis elegans*

## Abstract

In multicellular systems, cells communicate with adjacent cells to determine their positions and fates, an arrangement important for cellular development. Orientation of cell division, cell-cell interactions (i.e. attraction and repulsion) and geometric constraints are three major factors that define cell arrangement. In particular, geometric constraints are difficult to reveal in experiments, and the contribution of the local contour of the boundary has remained elusive. In this study, we developed a multicellular morphology model based on the phase-field method so that precise geometric constraints can be incorporated. Our application of the model to nematode embryos predicted that the amount of extra-embryonic space, the empty space within the eggshell that is not occupied by embryonic cells, affects cell arrangement in a manner dependent on the local contour and other factors. The prediction was validated experimentally by increasing the extra-embryonic space in the *Caenorhabditis elegans* embryo. Overall, our analyses characterized the roles of geometrical contributors, specifically the amount of extra-embryonic space and the local contour, on cell arrangements. These factors should be considered for multicellular systems.

## Abstract (precis)

**Summary:** The local contour and the extra-embryonic space, the empty space within the eggshell not occupied by embryonic cells, are important geometric constraints in cell arrangement of nematode embryos.

## INTRODUCTION

The arrangement of cells, which defines how cells come into contact with each other, is important in developmental processes. It mediates correct cell-to-cell communication and ultimately determines specific cell fates and body plans (Gilbert and Michael, 2019). This is a crucial homeostasis mechanism in multicellular organisms, including humans (Shahbazi, 2020). The mechanisms determining cell arrangement can be classified into three groups of factors: orientation of cell division; interactions (repulsion and attraction) between cells; and geometrical constraints provided by surrounding structures, such as the eggshell, in circumstances in which cells are confined (Baena-López et al., 2005; Gloerich et al., 2016; Schulze and Schierenberg, 2011; Yamamoto and Kimura, 2017). Numerical models including these factors have successfully explained cell arrangements during the early embryogenesis of *Caenorhabditis elegans* and sea urchins (Kajita and Yamamura, 2002; Akiyama et al., 2010; Fickentscher et al., 2013; Pierre et al., 2016; Yamamoto and Kimura, 2017; Giammona and Campas, 2021).

Among these three mechanisms determining cell arrangement, the effect of geometrical constraints has been the least explored. Most of the existing theoretical models of embryos have described eggshells as simplified spheres or ellipsoids, and no studies have reflected the precise shape of the eggshell. In contrast, real biological systems often contain local contours that are different from such simplified shapes. For example, the eggshells of nematodes are not perfect ellipsoids, in contrast to previous modeling efforts. Therefore, the development of a modeling framework that can incorporate complicated geometrical constraints is required. Such a framework is crucial for modeling cell arrangements with complicated geometrical constraints in tissues other than embryonic cells (Seirin-Lee, 2016). To this end, we decided to apply a phase-field method for the modeling of cell arrangement and directly reflected actual eggshell geometries in combination with imaging data. The phase-field method has the advantage of flexibly defining geometrical constraints (Akiyama et al., 2018; Nonomura, 2012; Moure and Gomez, 2021; Taniguchi et al., 2013; Seirin-Lee et al., 2020).

**Fig. 1.**
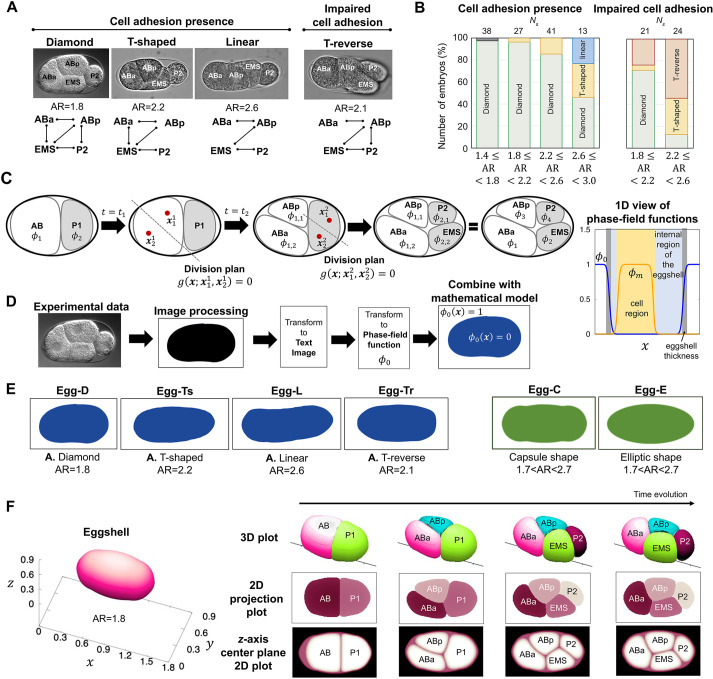
**Types of cell arrangement and cell morphology model using the phase-field method.** (A) Experimentally observed cell arrangements in four-cell-stage *C. elegans* embryos (images taken from Yamamoto and Kimura, 2017). Cell networks of the cell-to-cell contact states are shown beneath. ‘Impaired cell adhesion’ shows an embryo with knockdown of *hmr-1* and *hmp-2* genes, which are involved in cell adhesion. The normal (wild-type) embryo had an AR of ∼1.7, and embryos with a larger AR were obtained from mutation of the *lon-1* gene and/or knockdown of the *C27D9.1* gene. (B) Types of cell arrangements that appeared in each AR range. Green, yellow, blue, red and gray indicate diamond, T-shaped, linear, T-reverse, and pyramid-type arrangements, respectively. *N*_***s***_ indicates the sample number of embryos. Data derived from Yamamoto and Kimura (2017). (C) Schematic of the model. Red dots indicate the spindle poles, and the dotted line is the division plane determined by the location of the spindles. The one-dimensional view is shown on the right. (D) The process of incorporating the actual eggshell shape into a phase-field function. (E) Eggshell shapes used in numerical experiments. Egg-D, Egg-Ts, Egg-L and Egg-Tr were generated using the process shown in D with the actual eggshell experimental data from A. Egg-C and Egg-E were artificially generated using a phase-field model (Eqn 3; Materials and Methods). (F) Simulation example of the cell morphology model in three-dimensional space. The three-dimensional space data are shown by three different types of plots.

In this study, we chose the four-cell stage of nematode embryos as a biological model to study the precise contribution of geometric constraints to cell arrangement during embryogenesis. The four-cell stage of nematode embryos exhibits simple and diverse cell arrangements (Fig. 1A), which makes this stage a good target for comprehensively understanding the mechanisms underlying cell arrangement. The P_0_ cell refers to the one-cell stage after fertilization in *Caenorhabditis elegans*; this cell divides asymmetrically into two different daughter cells: the posterior P_1_ cell and anterior AB cell (two-cell stage). The AB cell first divides into anterior ABa and posterior ABp cells, and later, the P_1_ cell divides into anterior EMS and posterior P_2_ cells (Gönczy, 2005). As the P_2_ cell is adjacent to the ABp cell, but not to the ABa cell, the fate of ABp is distinct from that of ABa because of the signal from the P_2_ cell (Bowerman et al., 1992; Mickey et al., 1996).

In our previous study (Yamamoto and Kimura, 2017), we explored cell arrangement at the four-cell stage considering three factors: cell division orientation; repulsion and attraction between cells; and aspect ratios (ARs) of the ellipsoidal eggshell as a geometrical constraint. We succeeded in reproducing the four types of cell arrangement observed in different species and in *C. elegans* individuals with different ARs of the eggshell. The four types of cell arrangements were named pyramid, diamond, T-shaped and linear. The study showed that the asymmetric attraction between cells plays an important role in improving the robustness of cell arrangement, whereas eggshell AR is a source of diversity in cell arrangement, as shown in Fig. 1A,B. Meanwhile, our previous model failed to reproduce the T-reverse arrangement that appears when we elongated eggshells and impaired cell adhesion in the *C. elegans* embryo, suggesting that there is an important factor missing in our current understanding of cell arrangement.

In this study, using a modeling approach incorporating actual eggshell geometry by combining a phase-field function with imaging data, we found that the local contour is an important factor that can affect cell arrangement. Furthermore, from the successful reproduction of the T-reverse arrangement, we found that the quantitative contribution of the ‘empty’ space within the eggshell plays a crucial role in determining the cell arrangement. Here, the empty space refers to the space not occupied by the cells filled with an extra-embryonic matrix. We named this empty space the extra-embryonic space (ES). We demonstrated that the variability in the amount of ES can induce variability in cell arrangements even under similar conditions of cell division orientation, cell-cell interaction, and the AR of the eggshell. Moreover, we revealed that the effect of changing the ES can be modulated by controlling cell-cell interactions, such as surface tension and cell adhesion, and vice versa.

This finding provides a general concept that the amount of empty space can be a target for the regulation of cell arrangement, like the regulation of cell-cell interactions. This study proposes that, in addition to the global feature of geometric constraints such as AR, local features such as the local contour and the amount of ES play important roles in cell arrangement. These local features consequently result in the regulation of cell function.

## RESULTS

### Development of a cell morphology model incorporating the precise shape of the eggshell

To incorporate the precise geometry of the eggshell with the mathematical model, we developed a cell morphology model using a multiphase-field method (Fig. 1C,D). The major advantage of the phase-field method is its flexibility in describing shape (i.e. eggshell and cells). Using this to our advantage, we applied the model to input various shapes of the eggshell and successfully demonstrated the dynamic changes in cell arrangements as outputs. In this model, the eggshell and each cell are defined by phase-field functions, *φ*_0_(***x***) and *φ*_*m*_(***x***, *t*), respectively, where 0≤*φ*_0_(***x***)≤1 and 0≤*φ*_*m*_(***x***, *t*)≤1(*m*=1, …, 4) at location ***x***=(*x*_1_, …, *x*_*N*_), *N*=2, 3 and time *t*≥0, respectively (Fig. 1C, right panel). The interior of the eggshell is defined by {***x***|*φ*_0_(***x***)=0}, and the outside of the eggshell is defined by {***x***|*φ*_0_(***x***)=1}. The interior of the *m*th cell is defined by {***x***|*φ*_*m*_(***x***, *t*)=1}, and the outside of the cell is defined by {***x***|*φ*_*m*_(***x***, *t*)=0}. The region with 0<*φ*_*m*_(***x***, *t*)<1 corresponds to the cell membrane (border). In summary, the region occupied by the *m*th cell is defined as {***x***|*φ*_*m*_(***x***, *t*)>0}.

Each phase-field function *φ*_*m*_(***x***, *t*) (*m*=1, …, 4) describing cell dynamics is computed by solving the following equation, which is a general form of phase-field modeling:
(1)

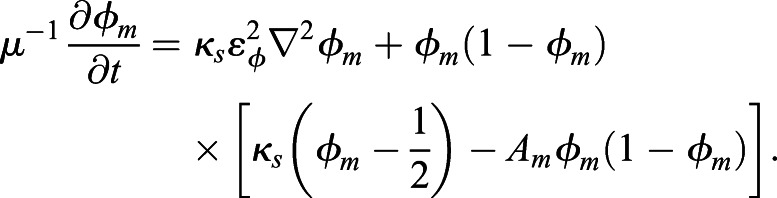
The basic formation of phase-field modeling is derived from the energy functional equations based on the Ginzburg–Landau free-energy equation (Provatas and Elder, 2010; Takagi and Yamanaka, 2012) (see Supplementary Materials and Methods for a detailed description of the energy functionals used for the modeling). In brief, *φ*_*m*_ changes over time to make the value of *A*_*m*_ zero, which is the equilibrium state. Therefore, the term *A*_*m*_ is the core part of phase-field modeling. In our cell morphology model, *A*_*m*_ (*m*=1, …, 4) is modeled as follows:
(2)

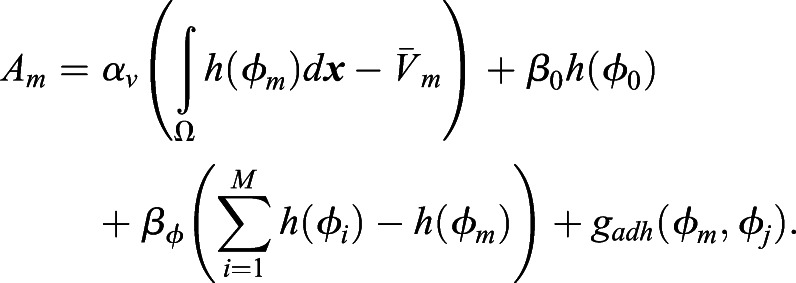
The first term of Eqn 2 defines the volume 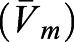 of the *m*th cell, and the second and third terms define the repulsive condition between cell and eggshell and between cells, respectively. The fourth term, *g*_*adh*_(*φ*_*m*_, *φ*_*j*_), defines the attraction between the *m*th cell and the *j*(≠*m*)th cell, and its value will reach zero when the *m*th cell and *j*th cell attract each other. *κ*_*s*_ denotes the surface tension parameter. *μ* and ε_*φ*_ are parameters defining the time scale of cell shape-changing dynamics and the thickness of the cell membrane, respectively (see Materials and Methods for full details of modeling). The function *h* is technically defined for the phase-field method (for details, see appendix of Seirin-Lee et al., 2016).

We extended the model of Eqns 1 and 2 to a data-combined model by defining *φ*_0_ with the actual eggshell shapes from image data (Fig. 1D). We also generated ellipsoidal or capsule eggshells using a phase-field model (see details in Materials and Methods). The various eggshell shapes used in the simulations are shown in Fig. 1E.

Using our cell morphology model, we first tested the dynamics of cell arrangement in three-dimensional simulations (Fig. 1F). From the test simulations, we concluded that the two-dimensional model is sufficient to examine the diamond, T-shaped, T-reverse and linear types of cell arrangement at the four-cell stage (Fig. 1A). This is because the orientation of cell division for the P_2_ cell is along the long axis of the egg, and that for the AB cell is perpendicular to the axis. As a result, the centers of the four cells of the four types of arrangement are roughly aligned on the common plane including the two axes (see two-dimensional projection plots and *z*-axis center plane plots of three-dimensional simulations in Fig. 1F). Thus, we mainly adopted a two-dimensional model rather than a three-dimensional model to incorporate the precise shape of the eggshell traced from the experimental data and to find unknown geometric effects by examining various parameter values with reduced numerical costs.

### Reproduction of the diamond, as well as T-shaped and linear, cell arrangements in the *C. elegans* four-cell-stage embryo

**Fig. 2.**
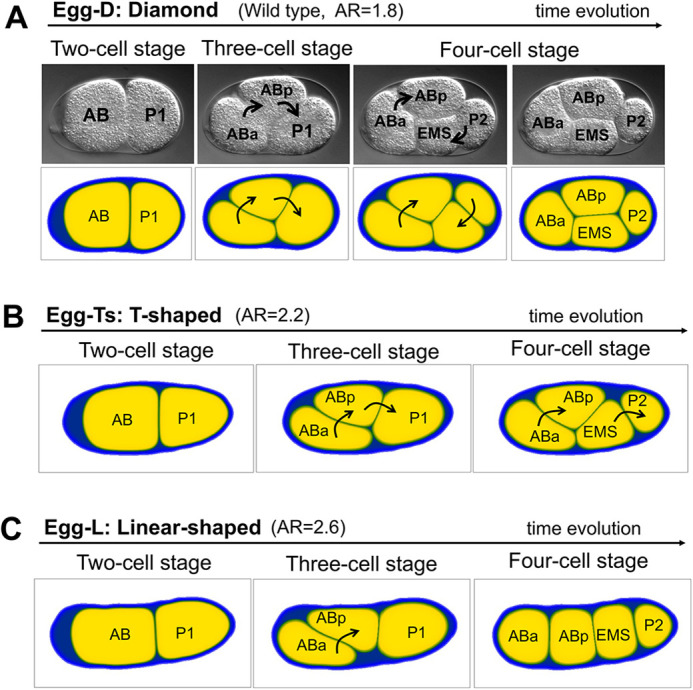
**Reproduction of representative cell arrangements by the cell morphology model with actual eggshell geometry.** (A) Representative data for the diamond arrangement of the wild-type embryo (upper panels; Movie 1) and simulations (lower panels; Movie 3). The embryo in the image is identical to that shown in figure S1 in Yamamoto and Kimura (2017). Cell adhesions exist. The eggshell shape was extracted from the image data of the upper panels and was the same as that of Egg-D. (B) Representative data for reproduction of the T-shaped arrangement. Cell adhesions exist. The eggshell shape was extracted from the image data shown in Fig. 1A, ‘T-shaped’, and is the same as Egg-Ts. (C) Representative data for reproduction of the linear arrangement. Cell adhesions exist. The shape of the eggshell was extracted from the image data shown in Fig. 1A, ‘Linear’, and is the same as Egg-L. Black arrows indicate the direction in which the cells rotate.

We first explored whether our cell morphology model accounts for the observed cell arrangement *in vivo*. We converted the eggshell of the real embryo into a phase-field function, *φ*_0_. We named the eggshell shape extracted from a wild-type embryo as Egg-D. Egg-D has an AR of 1.8, and shows diamond-type cell arrangement in the experiment. Eggshell shape from a *lon-1* mutation was named Egg-Ts, which has the AR of 2.2 and showed T-shaped arrangement. Similarly, Egg-L is the eggshell shape of a *C27D9.1* gene knockdown *lon-1* mutant embryos, with AR=2.6 that showed Linear arrangement (Fig. 1E). We then simulated how the cell arrangement changed from the two-cell to the four-cell stage (Fig. 2A), based on the model of Eqn 1. In a real embryo, after the division of AB and P_1_ cells, one of the cells (i.e. ABp cell) moves dynamically to adhere to the P_2_ cell and cells settle down into the diamond arrangement (Fig. 2A, upper panels; Movie 1). These behaviors were reproduced in our cell morphology model (Fig. 2A, lower panels; Movie 3).

We also tested eggshells Egg-Ts and Egg-L, and successfully reproduced T-shaped and linear arrangements, respectively (Fig. 2B,C). Of note, in this simulation, the T-shaped arrangement slowly changed to a linear type over a long period of time. Considering the rapid cell division that occurs during the embryogenesis of *C. elegans* (Yamamoto and Kimura, 2017), the T-shaped arrangement observed *in vivo* might be a transient state at the time of cell division, before it reaches the steady state of cell arrangement. Therefore, our model accounts for the cell arrangement observed *in vivo*.

### Reproduction of the T-reverse cell arrangement with the cell morphology model

**Fig. 3.**
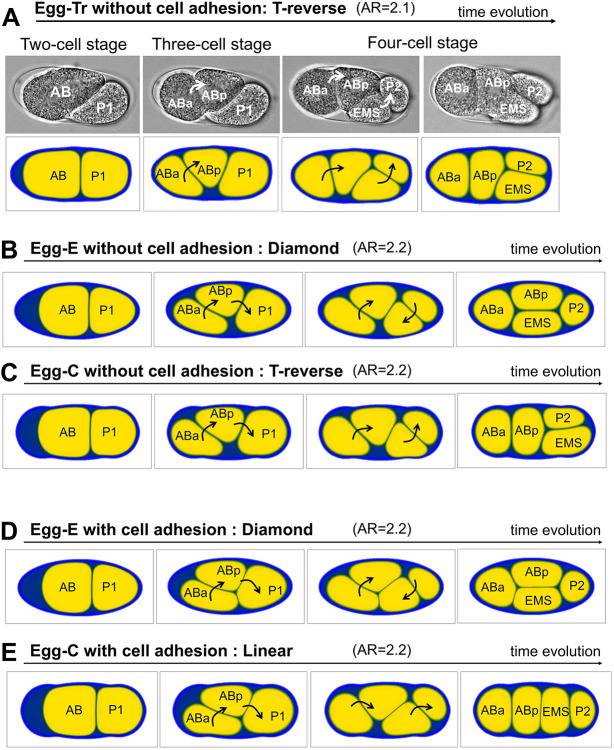
**T-reverse arrangement and the local contour effects of eggshell.** (A) Representative data for the T-reverse arrangement of the embryo (upper panels; Movie 2) and its simulations (lower panels; Movie 4). The experimental data were obtained from a *hmr-1; hmp-2*-double-knockdown embryo with a *lon-1(e185)* mutant background, in which cell adhesion was impaired. The embryo of the image is identical to that in figure 7C of Yamamoto and Kimura (2017). The eggshell shape was extracted from the image data shown above and is the same as that of Egg-Tr. (B,C) Numerical experiments for eggshells with the same AR (AR=2.2) but different shapes when cell adhesion is absent. (D,E) Numerical experiments for eggshells with the same AR (AR=2.2) but different shapes when cell adhesions are present. Black or white arrows indicate the direction in which the cells rotate.

Next, we investigated whether we could reproduce the T-reverse-type arrangement using the cell morphology model. In our previous experiment (Yamamoto and Kimura, 2017), a T-reverse arrangement was found in the *hmr-1; hmp-2*-double-knockdown condition with a *lon-1(e185)* mutant background. This indicates that cell adhesion was impaired in an elongated eggshell (Fig. 3A, upper panels; Movie 2). In the T-reverse arrangement, the ABp cell moves toward the posterior, whereas the movement of the EMS cell is smaller and it does not adhere to the ABa cell. However, our previous model, which assumes an ellipsoidal eggshell, did not account for the T-reverse arrangement (Yamamoto and Kimura, 2017).

In the cell morphology model, we extracted the eggshell shape from a *lon-1* mutant embryo with an AR of 2.1, named Egg-Tr (Fig. 1E) and simulated the condition of the T-reverse arrangement (i.e. Egg-Tr without cell adhesion). Surprisingly, the T-reverse arrangement was successfully reproduced in our first trial (Fig. 3A, lower panels; Movie 4). This is the first example of successful reproduction of the T-reverse arrangement in a mathematical model.

In the *C. elegans* embryo, the T-reverse arrangement was not observed in the wild type, but in an elongated eggshell without cell adhesion. However, in related nematode species the T-reverse arrangement has been observed in the wild type (Dolinski et al., 2001). We confirmed the appearance of a T-reverse arrangement in the *Cephalobus* sp. (DWF1301 strain) (Fig. S1A, Movie 5). Moreover, our cell morphology model incorporating the actual shape of the eggshell, cell size, and cell division orientation of *Cephalobus* sp. successfully reproduced the T-reverse arrangement (Fig. S1B). Therefore, our reproduction of the T-reverse arrangement is an important step forward in understanding the mechanics underlying the diversity of cell arrangement among species.

### The importance of the local contour of the eggshell on the cell arrangement

Why were we able to reproduce the T-reverse-type arrangement when our previous model (Yamamoto and Kimura, 2017) could not? Most theoretical studies, including our previous work (Yamamoto and Kimura, 2017), assume an elliptical shape of the eggshell (Kajita and Yamamura, 2002; Pierre et al., 2016; Giammona and Campas, 2021). However, the actual shape of the eggshell, which we incorporated into the current model, is similar to a capsule, that is, a tube with two ends covered by two hemispheres. To test whether the difference between elliptical and capsule shapes was crucial for reproduction of the T-reverse type, we compared the elliptical (Egg-E) and capsule (Egg-C) shapes of eggshells with the same AR (2.2) and without cell adhesion (Fig. 3B,C). We found that the capsule shape induces a T-reverse arrangement, whereas the elliptical shape induces a diamond arrangement. In the case of a capsule shape, the eggshell space in the posterior edge is wider than that of an elliptical shape, and therefore, EMS and P_2_ do not need to rotate much to align with the long axis of the eggshell, consequently leading to a T-reverse arrangement. This result suggests that the precision of eggshell geometry is crucial for cell arrangement.

Thus, we investigated whether the local contour can affect cell arrangement even when cell adhesion exists. We found that the capsule eggshell with AR=2.2 has a linear arrangement, whereas the elliptical eggshell of the same AR has a diamond arrangement (Fig. 3D,E). In summary, these results demonstrate that the local contour, in addition to the global feature of the AR, is involved in determining cell arrangements.

### The effect of extra-embryonic space on cell arrangement

Why did local contour affect cell arrangement? The contour of the capsule shape provided more space for cells to move. Thus, we examined the ES within the eggshell; that is, the inner space of eggshell that is not occupied by the cells but filled with extra-embryonic matrix.

**Fig. 4.**
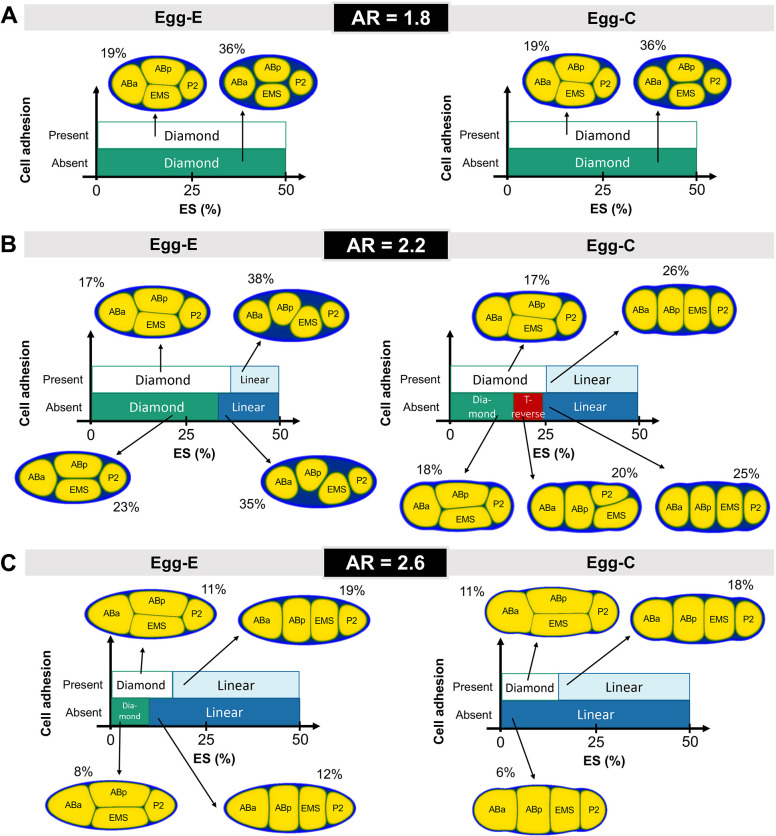
**The effect of the ES ratio and eggshell geometry in cell arrangement.** (A-C) Types of cell arrangement depending on the scale of the ES, which was calculated by [1−(Total area of cells/Internal area of eggshell)](%). The representative simulation results are shown for each case, and the marked percentages indicate the amount of ES in each schematic example.

We systematically investigated how the ratio of ES affects the asymptotic cell arrangement with respect to several scales of the AR (Fig. 4). ES was calculated as follows: 1−(total sum of cell areas/internal area of eggshell) (expressed as a percentage). See ‘Extraction of the actual eggshell shape, ES calculation and parameter values’ section in Materials and Methods for the detailed mathematical definition of ES. Thus, ES was regulated by changing the target volume parameter 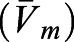 in Eqn 1 while maintaining the relative cell volume and fixing the other kinetic parameters. Furthermore, to determine the influence of sensitivity to eggshell shape, we also compared elliptical (Egg-E) and capsule (Egg-C) eggshells under the same AR conditions, both with and without cell adhesion. We found that ES plays an important role in diversifying cell arrangements. Our findings on the contribution of ES to cell arrangement are summarized below.

First, the simulation results demonstrated that the amount of ES causes diversity in cell arrangement, even with the same AR. Let us focus on Egg-C with AR=2.2 in the absence of cell adhesion (Fig. 4B, Egg-C, Absent), which is similar to the experimental conditions of the *lon-1; hmr-1; hmp-2* mutant/knockdown (Fig. 1A, ‘T-reverse’, 1B, right). The simulation predicted three types of arrangements (diamond, T-reverse and linear) depending on the ES ratio. We also confirmed the same results in a three-dimensional model (Fig. S2). We confirmed whether the ES ratio can change the cell arrangement, as shown in the 2D simulation results of Fig. 4. We generated a capsule eggshell with AR=2.2 and set the ES ratios to 23%, 27% and 32%. The results of Fig. S2 show that a diamond arrangement is generated as ES is small, and the T-reversed and linear arrangements appear in sequence as we increase the ratio of ES. This result directly demonstrates that diverse arrangements can be reproduced by changing the amount of ES within embryos with the same AR.

Second, we found that the sensitivity of the cell arrangement to the ES ratio depends on the AR. When the AR was 2.2, as explained in the previous paragraph, the cell arrangement was sensitive to the ES ratio (Fig. 4B). However, when the AR was 2.6, the sensitivity to the ES ratio decreased. In both elliptical and capsule eggshells with AR=2.6, the embryos showed a linear arrangement over a wide range of ES ratios, and the ES ratio generated a diamond arrangement that was very restricted (Fig. 4C). When the AR of the embryo was 1.8, the sensitivity to the ES was lost and the embryos occupied the diamond arrangement regardless of the ES ratio, at least for the Egg-E and Egg-C shapes that we examined (Fig. 4A). Interestingly, we noted a T-reverse arrangement for the Egg-D shape with an ES ratio of 32% and without cell adhesion (Fig. S3, as shown in Fig. 6C). This is another demonstration that the precise contour of the eggshell affects cell arrangement. Furthermore, the arrangement remained diamond when adhesion was present, even in the Egg-D shape with an ES ratio of 32% (Fig. S3). This demonstrates that the function of cell adhesion is to increase the robustness of cell arrangement against the precise cell contour, in addition to the robustness against the AR, as we proposed previously (Yamamoto and Kimura, 2017).

Finally, we noticed that the effect of cell adhesion was also sensitive to ES. By comparing the presence or absence of cell adhesion, we found that cell adhesion could affect cell arrangement only within certain ranges of the ES ratio. For example, for Egg-C and AR=2.2 (Fig. 4B, right), the loss of cell adhesion changed the cell arrangement from the diamond or linear arrangements to the T-reverse when the ES was between ∼ 19 and ∼ 24%. In contrast, the cell arrangement remained unchanged regardless of cell adhesion for ES<∼19% and ES>∼24%.

Considering these results, we conclude that the amount of ES can be a crucial factor in determining cell arrangement. The cell arrangement was sensitive to the amount of ES, and the effect of cell adhesion on cell arrangement was also sensitive to ES. Meanwhile, the magnitude of sensitivity to cell arrangement with respect to the ES, eggshell shape (i.e. elliptical or capsule) and cell adhesion depended on the AR of the eggshell. Therefore, the combined effect of ES and AR underlies the cell arrangement. Moreover, changes in the amount of ES might be a source of the variability in cell arrangement observed for various species of nematodes (see below, section ‘The amount of ES as the cause of diversity in cell arrangement’).

### Experimental evidence that the amount of ES affects cell arrangement

**Fig. 5.**
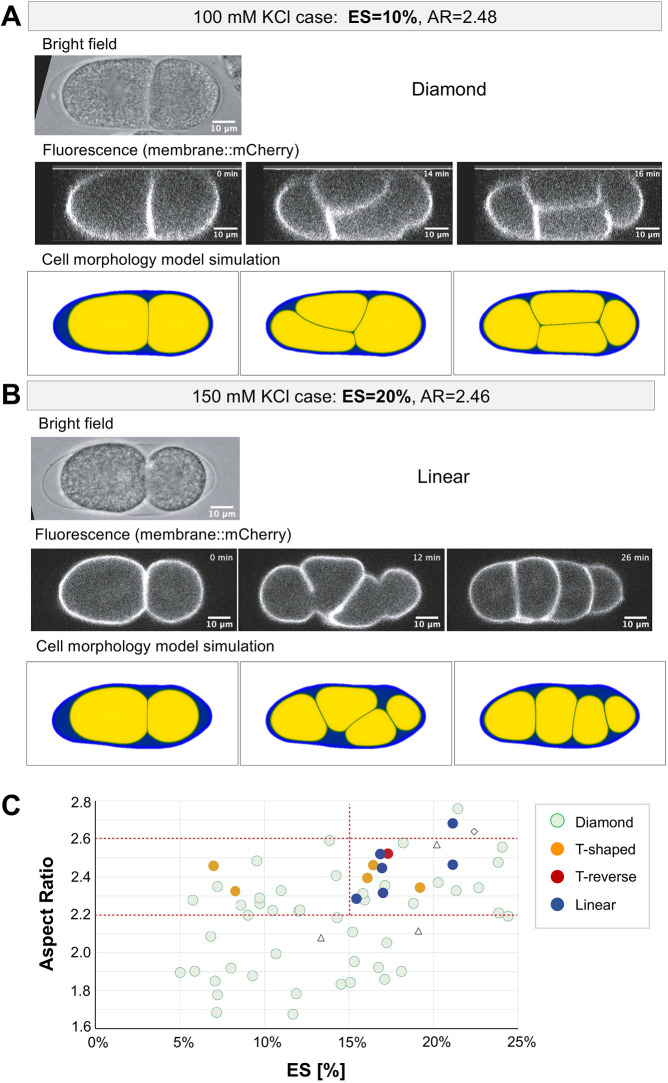
**Experimental manipulation of the amount of ES, and our cell morphology model simulation for the corresponding situation.** (A) Top: A brightfield microscopy image of a representative embryo of CAL2152 (*lon-1*; PH::mCherry) with *perm-1; C27D9.1* (RNAi) in 100 mM KCl solution at the two-cell stage. The AR was 2.48 and the ES was 10% for this embryo. Middle: Time-lapse images obtained by fluorescence confocal microscopy. The elapsed time from the beginning of imaging is indicated. To represent the cell arrangement better, a plane perpendicular to the confocal sections is shown after an optical three-dimensional reconstruction. Bottom: Cell morphology model simulation with the actual eggshell shown in the brightfield image with an ES of 10%. (B) As in A, except that the embryo was mounted in 150 mM KCl solution. As a result, ES increased to 20% for this embryo. For the fluorescence images, confocal sections are shown, without three-dimensional reconstitution. (C) Summary of the results for *perm-1* (RNAi) embryos treated either with 100 mM or 150 mM KCl solution. The amount of ES, AR, and the resultant cell arrangement are shown as green (diamond), blue (linear), orange (T-shaped), red (T-reverse) circles. Embryos with a wide variety of ARs were obtained by using the *lon-1* mutation with *C27D9.1* (RNAi) in addition to the untreated (control) individuals. In some cases (*n*=4/61, unfilled triangles and rhombus), the orientations of the cell division were both perpendicular to the long axis, and the timing of the division was almost simultaneous. These four cases were excluded from later analyses.

We next aimed to obtain experimental evidence that ES affects cell arrangement. To increase the amount of ES in the *C. elegans* embryo, we utilized RNAi knockdown of the *perm-1* gene. Knockdown of *perm-1* increases the permeability of eggshells (Carvalho et al., 2011). Otherwise, no apparent defects in early embryogenesis have been reported, although embryogenesis halts before hatching. Placing *perm-1* (RNAi) embryos in a 150 mM KCl solution led to an increase in the amount of ES compared with that in wild-type cells or *perm-1* (RNAi) embryos in 100 mM KCl (Fig. 5A,B, brightfield). Under these conditions, the embryos showed a linear arrangement (Fig. 5B, fluorescence; Movie 7), in contrast to the diamond arrangement in the presence of less ES (Fig. 5A, fluorescence; Movie 6). This provides direct experimental support that the amount of ES changes the cell arrangement. This experimental observation agrees well with the prediction made by our cell morphology model that the linear arrangement will appear when we increase the amount of ES in elongated eggshells (Figs 4B,C, 5A,B simulations).

**Fig. 6.**
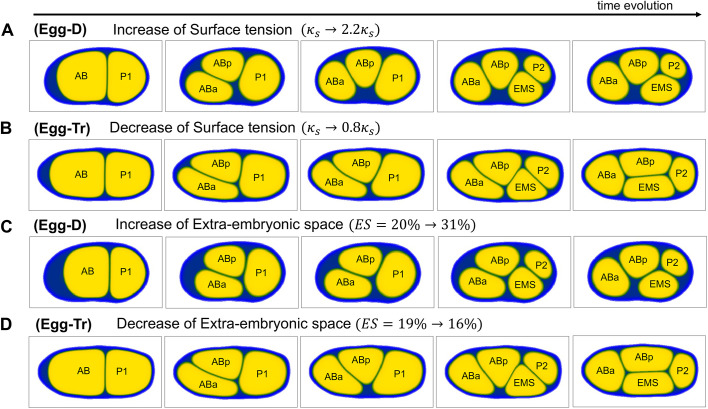
**Surface tension and ES.** (A-D) Simulations not including cell adhesions. (A) Egg-D (AR=1.8) simulation with increased surface tension (κ_*s*_). ES=28%. (B) Egg-Tr (AR=2.1) simulation with decreased surface tension (κ_*s*_). ES=18%. (C) Egg-D (AR=1.8) simulation with increased ES. The ES increased from 20% to 31%. (D) Egg-Tr (AR=2.1) simulation with decreased ES. The ES decreased from 19% to 16%.

By repeating the observations, we analyzed the relationship between ES, aspect ratio, and cell arrangement for 62 embryos (Fig. 5C). Four embryos appeared to have abnormalities in cell division orientations and were excluded from the analyses. All embryos with ARs below 2.2 (*n*=22) exhibited the diamond arrangement. This result agrees with our prediction that the diamond arrangement is dominant regardless of the ES at AR=1.8, or with ES<25% at AR=2.2 (Fig. 4A,B). The probability of obtaining the diamond arrangement (*n*=46/58 for total observations) for all 22 embryos with AR<2.2 by chance was *P*=0.001 for the binomial test. This calculation supports our argument from a statistical viewpoint.

For a higher AR, AR>2.2, we observed non-diamond arrangements in an ES-dependent manner, as predicted by our model. Of the 12 non-diamond embryos, ten embryos exhibited *ES*>15%. The probability of this by chance is *P*=0.047, indicating that the amount of ES affects the cell arrangement. In a previous analysis of embryos with normal ES and AR=2.2-2.6, a T-shaped arrangement was observed for 15% of the embryos for AR=2.2-2.6 (Yamamoto and Kimura, 2017) (Fig. 1B). This rate was reproduced in the present observation, in which two out of 14 embryos showed a non-diamond (T-shape) arrangement for embryos with ES<15% and 2.2<AR<2.6. In contrast, almost half of embryos (*n*=9/20) with ES≥15% and 2.2<AR<2.6 showed non-diamond arrangements. This rate of the non-diamond arrangement was significantly higher than that of normal ES embryos (*P*=0.001 with the binomial test). This comparison also indicates that the amount of ES affects cell arrangement.

In particular, we observed five embryos with a linear arrangement with AR within the range 2.2-2.6 and ES≥15%. This is in contrast with our previous observation for a normal amount of ES (Yamamoto and Kimura, 2017), in which we never observed a linear arrangement for the same AR (Fig. 1B). This contrast observation for low and high ES agrees with the prediction from our cell morphology model that the linear arrangement is expected with a larger ES for AR=2.2 and 2.6 (Fig. 4B,C).

The experimental results showed variability such that the diamond arrangement was still observed in the high ES and high AR range. This variability may be due to variability in cell division axes or cell-cell interactions. Despite the variability in the experimental results, we argue that the predictions from the cell morphology model accurately describe the major features of the experimental observations.

The present experimental treatment may not only affect the cell size, but also the physical properties of the cell, such as surface tension. Further evaluation of such parameters will be important in the future. Meanwhile, from our analyses introduced in the next section, we know that the change in parameters of physical properties has a similar effect on ES and the cell arrangement as the control in cell size. Therefore, our experimental correlation between the ES amount and cell arrangement supports our model in the end, regardless of the change in ES induced by the control in cell size or a physical parameter such as the surface tension.

### The change in ES amount had similar effect to the control of surface tension

To see how the physical properties of each cell influence the cell arrangement, we focused on the parameter of cell surface tension (*κ*_*s*_) in our model of Eqn 1 and changed it to different scales. From the simulation tests, we found that the T-reverse arrangement could be obtained when the cell surface tension was increased, even in the eggshell shape of the wild type (Egg-D) (Fig. 6A). Furthermore, we found that the diamond arrangement can be obtained when the cell surface tension was decreased, even in the eggshell shape of the T-reverse arrangement (Egg-Tr) (Fig. 6B). We presumed that cell surface tension was likely to be effective when the cells were not compressed within the eggshell. These results are also consistent with our conjecture that ES affects cell arrangement.

We tested whether the amount of ES and the surface tension have similar effects on cell arrangement by changing the amount of ES in the model under the condition of fixed surface tension. We found a T-reverse arrangement when we increased the ES by decreasing the volume of cells but maintaining the relative volume (Table S1) between the cells in the eggshell of wild type (Fig. 6C). Similarly, we found a diamond arrangement when we decreased the ES and compressed the cells in eggshells of Egg-Tr (Fig. 6D). The results indicate that the amount of ES plays a role similar to that of the control of surface tension, which affects cell arrangement.

### The amount of ES as the cause of diversity in cell arrangement

Through this study, we revealed that the cell arrangement varies depending on the amount of ES, even under a fixed cell division orientation, cell-cell interaction, and AR of the geometric constraint. This raises an important message that we should consider the state of the ES (or equivalent empty spaces) when assessing the mechanisms underlying the cell arrangements.

We found that a change in the amount of ES had a similar effect to a change in cell surface tension (Fig. 6). Interestingly, the results indicated that the extracellular environment and cell-autonomous activity play an interchangeable role in cell arrangement. The nematode species show diverse cell arrangements even if they have an AR similar to that of the eggshell (Yamamoto and Kimura, 2017). Previously, we speculated that this difference was caused by differences in cell adhesion/tension (Yamamoto and Kimura, 2017). The present study provides another possibility that a change in the amount of ES causes diversity among species.

**Fig. 7.**
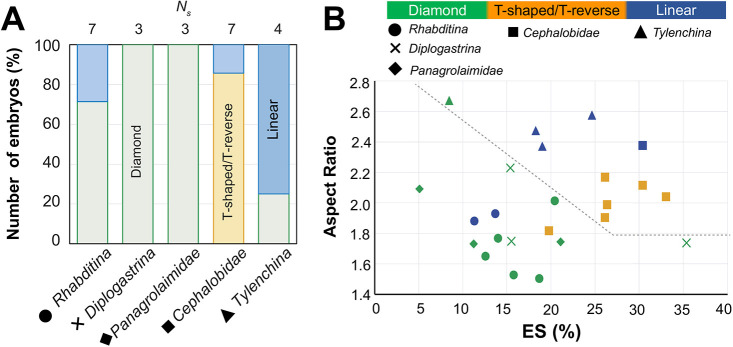
**Analysis of cell arrangement types and ES in several families of nematodes.** Image data of eggshells with 1.5<AR<2.7 from Dolinski et al. (2001) was used for the analysis. *Rhabditina* (*N*_***s***_=7), *Diplogastrina* (*N*_***s***_=3), *Panagrolaimidae* (*N*_***s***_=3), *Cephalobidae* (*N*_***s***_=7), *Tylenchina* (*N*_***s***_=4). (A) The ratio of cell arrangement types for several families of nematodes: diamond (green), T-shaped/T-reverse (orange) and linear (blue). (B) Classification of embryos for each nematode family by ES ratios and AR. The different colors indicate different cell arrangement types, as shown in A. Different symbols indicate different families, as indicated in the key. The gray dotted lines were drawn by hand as a boundary separating diamond-predominant families (circle, cross, rhombus) and non-diamond-predominant families (square, triangle), except for one species at ES=20%, AR=1.8.

We next examined the amount of ES in other nematode species (Fig. 7). We investigated the cell arrangement types in five families from a previously published study (Dolinski et al., 2001) (Fig. 7A), indicating that different families have different predominant cell arrangement types. *Rhabditina* (1, circle), *Diplogastrina* (2, cross) and *Panagrolaimidae* (3, rhombus) predominantly show the diamond arrangement (green). In contrast, *Cephalobidae* (4, square) and *Tylenchina* (5, triangle) predominantly show T-shaped/T-reversed (orange) and linear (blue) arrangements, respectively. Interestingly, when we plotted the ARs of these species against the ES, we found that the three families with a predominantly diamond arrangement (1-3) had lower ES and AR than the two species with predominantly non-diamond arrangements (4, 5) (Fig. 7B, gray dotted line). This observation indicates that a higher ratio of ES is observed for species favoring T-shaped, T-reverse or linear arrangements, even within the same AR range (for example, 1.8<AR<2.2 in Fig. 7B). In other words, both the AR and the ES differ across species and they both contribute to defining the cell arrangement of the species. We propose that the ES ratio is an important measure to induce different cell arrangements in different species of nematodes.

Moreover, the prediction of the model (Fig. 4) is applicable for understanding the diversity among species. First, all species with a low AR (≤1.8) take the diamond arrangement. This concentration of the diamond arrangement in the low-AR region is statistically relevant (*P*=0.0007). This agrees with the prediction of our model that embryos will take the diamond arrangement at AR=1.8 regardless of the ES and the strength of cell adhesion (Fig. 4A), and with our experiments using *C. elegans* (Fig. 5C). This observation supports the idea from our cell morphology model that AR=1.8 provides robustness for the diamond arrangement against variability in the strength of cell adhesion and the amount of ES, and thus some species adopt this AR in nature.

Next, we observed the non-diamond (i.e. T-shaped, T-reverse and linear) arrangements for higher AR (>1.8) in a manner dependent on the amount of ES. The diamond arrangement (Fig. 7B, green symbols) is more common in the lower ES range than in the non-diamond arrangements (Fig. 7B, blue and orange symbols). This argument is supported by statistical testing. Among the 16 species with AR>1.8, four occupy the diamond arrangement. Among the four diamond species, two were concentrated in the lowest two species out of the 16 species in total (*P*=0.05), three in the lowest five (*P*=0.06) and four in the lowest nine (*P*=0.07) (Fig. 7B). These observations support our cell morphology model, suggesting that ES is important to define cell arrangement for diverse species in nematodes.

It should be noted that although the amount of ES is important, it is not the sole determinant of cell arrangement. The correlation between the amount of ES and cell arrangement is not perfect, as shown in Fig. 7. Diversity among species might be caused by a combination of other factors, such as cell division orientation and cell-cell interactions.

## DISCUSSION

### Construction of cell morphology model by using the multiphase-field method

The application of the multiphase-field model for multicellular systems was introduced by Nonomura (2012), and this modeling method has been applied to various biological phenomena (Akiyama et al., 2018; Moure and Gomez, 2021; Seirin-Lee et al., 2020; Seirin-Lee, 2016). In this study, we aimed to investigate the role of spatial constraints in the cell arrangement of *C. elegans* embryos from a geometrical viewpoint. For this purpose, we adopted the phase-field method. We traced the actual eggshell shape of the *C. elegans* embryo and incorporated it into the phase-field model. The phase-field model is advantageous for describing the precise shape of the eggshell; thus, we did not need to approximate the shape by using a simplified shape such as an ellipsoid.

In this study, we mainly adopted a two-dimensional model rather than a three-dimensional model, to incorporate the precise shape of the eggshell traced from the experimental data and to find unknown geometrical effects by examining various parameter values with reduced numerical costs. However, the general mathematical formulation of phase-field modeling is written as an *N*-dimensional spatial format, so that extension to higher-dimensional modeling is possible once the obstacle of numerical costs is solved. In this study, we confirmed that three-dimensional models and two-dimensional models give consistent results for the cell arrangements in the four-cell stage (Fig. 1, Fig. S2). The two-dimensional model was sufficient to explore the diamond, T-shaped, T-reverse and linear types of cell arrangement in this study. However, for other cases, such as the pyramid-type arrangement or arrangement in later stages, three-dimensional modeling is required (Moure and Gomez, 2021; Saito and Sawai, 2021; Ansari et al., 2021).

### Reproduction of the T-reverse type cell arrangement

The model that we constructed previously (Yamamoto and Kimura, 2017) was named the asymmetric attraction (AA) model, based on vertex mechanistic dynamics between the mass points of cells. Although the AA model explains the robustness of the diamond-type arrangement against the change in the AR of the eggshell, there were some inconsistencies between the mathematical model and the actual embryos. The AA model predicted the linear-type arrangement in elongated eggshells without the attraction force, but the actual embryos showed a T-type or T-reverse-type arrangement (Yamamoto and Kimura, 2017). The AA model cannot reproduce the T-reverse arrangement. Using our cell morphology model, we succeeded in reproducing the T-reverse-type arrangement (Fig. 3A, Fig. S2). Incorporation of the actual eggshell shape was a key factor (Fig. 3). In addition, we found that the amount of ES was crucial for reproducing the T-reverse arrangement (Fig. 4).

### The role of geometric constraints in the determination of cell arrangements

Cell arrangement is important for development and is known to be determined by three factors: the orientation of cell division, strength of cell-cell interactions (i.e. attraction and repulsion between adjacent cells), and geometric constraints. Compared with the first two factors, investigations on geometric constraints have been limited. Previously, we demonstrated the contribution of the AR, which is a global feature of geometric constraints (Yamamoto and Kimura, 2017). In this study, we demonstrated the contribution of empty space, which is a local feature of geometric constraints. The empty space is the ES in the *C. elegans* embryo. We believe that the state of empty space is also important for cell arrangement in other cell types of cell stages later than the four-cell stage, and in species other than *C. elegans*, and in non-nematode species. This study is currently in progress. It is important to note that our model can easily change the rigidity of the boundary condition, i.e. the eggshell, in this study. Therefore, our model is applicable to the cell arrangement of embryos in soft membranes, or a group of cells surrounded by other cells or soft tissues.

Although we focused on the local contour of the eggshell and the amount of ES in this study, our cell morphology model should provide a powerful means to examine the effect of the orientation of cell division, strength of cell-cell interactions and the magnitude of stochastic fluctuations, in addition to that of geometric constraints. Importantly, these factors affecting cell arrangements can also affect each other. Although the orientation of cell division is primarily determined by the orientation of the mitotic spindle, the orientation of the spindle may skew during the elongation of the spindle before cell division. This alteration is also affected by the local contour of the eggshell and the amount of ES, which may affect how we define cell division in the mathematical model. Thus, further investigation is needed to integrate the interplay of all factors. The present cell morphology model provides a general mathematical formulation that can integrate further factors.

## MATERIALS AND METHODS

### Multiphase-field method

To describe the shapes of cells precisely, we chose phase-field modeling. The technical application of the phase-field method for the morphodynamics of single cells was introduced by Shao et al. (2010, 2012). The multiphase-field method applied to multicellular systems has been well described by Nonomura (2012), and its applications to pattern formation in multicellular systems and nuclear chromatin dynamics have been demonstrated by Seirin-Lee and colleagues (Seirin-Lee, 2016; Seirin-Lee et al., 2016, 2020). In this study, we constructed a mathematical model describing the transformation from the two-cell stage to the four-cell stage of the *C. elegans* embryo using the multiphase-field method introduced by Nonomura (2012), Seirin-Lee et al. (2016) and Akiyama et al. (2018). Note that the phase-field model description is basically written in the *N*-dimensional spatial case, and there is no difference in the formulation of modeling between high-dimensional cases.

### Multicellular morphology model of the *C. elegans* embryo

We designed the model with phase-field functions defined by eggshell, *φ*_0_(***x***), and daughter cells derived from the fertilized mother cell, *φ*_*m*_(***x***, *t*)∈[0, 1] (1≤*m*≤*M*), where ***x***∈Ω in ***R***^*N*^, *t*>0, *M* is the total number of cells, and Ω denotes the system area. In the two-cell stage, *M*=2 and the AB and P_1_ cells are defined by the first cell (*m*=1) and second cell (*m*=2). In the four-cell stage, *M*=4 and ABa and EMS cells are defined by the first cell (*m*=1) and second cell (*m*=2), ABp and P_2_ cells are defined by the third cell (*m*=3) and fourth cell (*m*=4), respectively (Fig. 1C). The region of the cell is defined as {***x***|0<*φ*_*m*_(***x***, *t*)≤1}. We also define the region of eggshell as {***x***|0<1−*φ*_0_(***x***)≤1} by defining the internal area of the eggshell as {***x***|1−*λ*_0_<1−*φ*_0_(***x***)≤1}, where *λ*_0_ is the value determining the thickness of eggshell (see Discussion for more detail).

A detailed model description using energy functionals is described in Supplementary Materials and Methods. Here, we briefly describe the transformed evolutionary system. Through transformation from the energy functionals to the evolutionary system (Supplementary Materials and Methods), the model equation of the eggshell and the time evolution of the *m*th cell are given by the following equations:
(3)

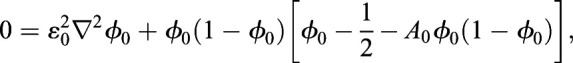

(4)

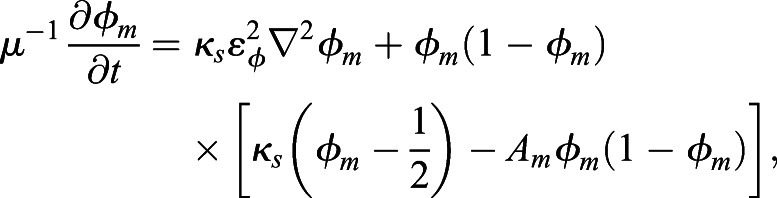
where *μ*_0_, *μ*, ε_0_, ε_*φ*_ are positive constants, and *κ*_*s*_ is the surface tension. *A*_0_ and *A*_*m*_ are given by:
(5)

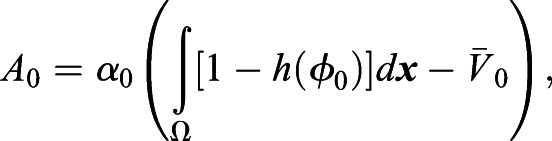

(6)

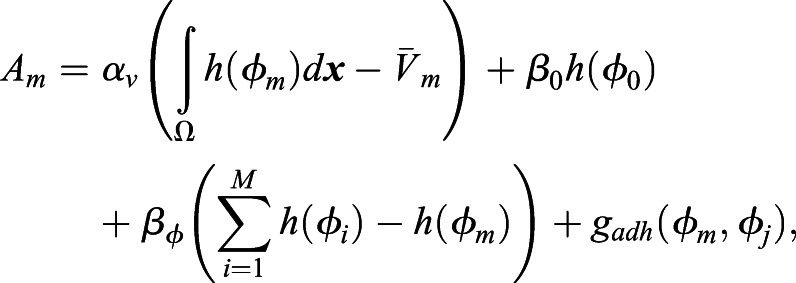
where *α*_0_, *α*_*v*_, *β*_0_, *β*_*φ*_ are positive constants. 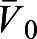 denotes the volume of the eggshell. 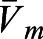 is the volume of the *m*th cell, and *h*(*φ* )=*φ*^3^(10−15*φ*+6*φ*^2^). The first terms of *A*_0_ and *A*_*m*_ define the volumes of the eggshell and the *m*th cell. The second and third terms of *A*_*m*_ define the territory (repulsive) condition in which the cell regions do not overlap. The fourth term, *g*_*adh*_(*φ*_*m*_, *φ*_*j*_), of *A*_*m*_ defines the attraction between the *m*th cell and *j*(≠*m*)th cell and is given as:
(7)

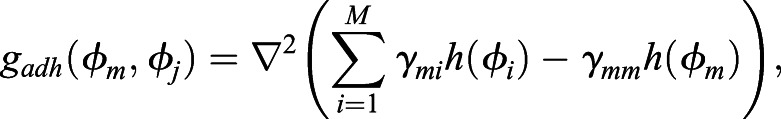
where *γ*_*mi*_ is the strength of attraction between the *m*th cell and the *i*th cell such that *γ*_*mi*_=*γ*_*im*_. As the ABa and P_2_ cells are not in contact in the four-cell stage, we set *γ*_14_=*γ*_41_=0. Based on the experiment of Yamamoto and Kimura (2017), We assume that the attractions, EMS–P_2_ (*γ*_24_) < ABa–ABp (*γ*_13_) < ABa–EMS (*γ*_12_) ≈ ABp–EMS (*γ*_23_). Although the eggshell removal experiment did not directly provide information on ABp–P_2_ (*γ*_34_), we estimated *γ*_34_ ≈ *γ*_24_ based on the localization of the cadherin molecule, and the estimation reproduced well the cell arrangements under various conditions (Yamamoto and Kimura, 2017).

### Cell division in sequence

The direction and position of the cell division plane are important elements that can affect the initial cell position. Spindle positioning, which determines the location of the cell division plane, is tightly regulated by both biochemical and biophysical dynamics (Coffman et al., 2016; Cheng et al., 1995; Kimura and Onami, 2007; Minc et al., 2011; Manuel Théry et al., 2007). In our model, we fixed the direction and position of the cell division plane based on the image of the wild-type *C. elegans* embryo, unless indicated. We previously confirmed that the *lon-1(e185)* mutation and RNAi knockdown of *C27D9.1*, *hmr-1* and *hmp-2* genes did not affect the orientation of cell division (Yamamoto and Kimura, 2017). *perm-1* (RNAi) also did not affect the cell division orientation in most cases, as reported by Carvalho et al. (2011). In some cases, however, we detected changes in the cell division orientation (*n*=4/62; Fig. 5C, triangles and diamond). We excluded minor cases from further analyses.

Thus, we considered the simplest model in which the direction and position of the cell division plane is determined by the plane bisecting the one connecting the given spindle poles at the center (Fig. 1C) (Akiyama et al., 2018). Let us set the spindle poles of the *m*th cell as 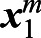 and 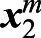 and the divided daughter cells of the *m*th cell, *φ*_*m*_, by *φ*_*m*,1_, and *φ*_*m*,2_. Then the daughter cells are described as:
(8)

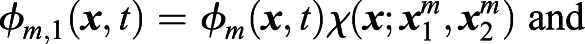

(9)

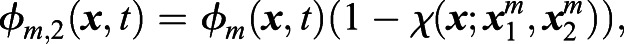
where
(10)

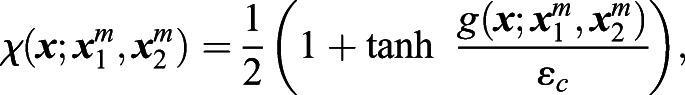
and ε_*c*_ is a positive constant. The function of *χ* is a step function of one or zero depending on the region bisecting the division plan, 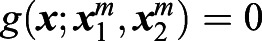. The function of 
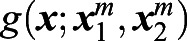
 is defined as:
(11)

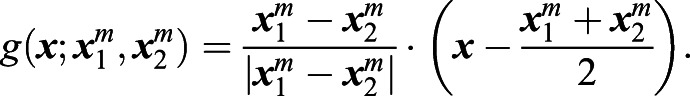
We started simulations with the two-cell stage of the AB cell (*φ*_1_) and P_1_ cells (*φ*_2_). When *t*=*t*_1_, we first divided the AB cell into the ABp cell (*φ*_1,1_) and ABa cells (*φ*_1,2_). Then, we divided the P_1_ cell into P_2_ cell (*φ*_2,1_) and EMS cell (*φ*_2,2_) when *t*=*t*_2_(>*t*_1_). After cell division, we replaced the phase-field functions with 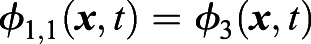, 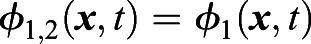, 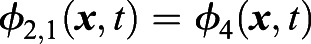 and 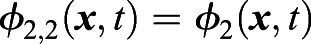.

### Extraction of the actual eggshell shape, ES calculation and parameter values

To investigate the geometric effect of the eggshell precisely, we simulated a model (Eqn 4) with 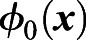 that reflects the actual shape of the *C. elegans* eggshell by converting the images from the experiment to numeric data (Fig. 1D). Conversion of the eggshell image to numeric data and imaging analysis was performed using ImageJ (Version:2.1.0/1.53c).

The eggshell volume was fixed for all simulations, and the volume of each cell was determined based on the experimental data (Table S1, Fig. S4). ε_0_ and ε_*φ*_ were estimated from the thickness of the eggshell (*δ*_0_=0.4 μm) (Krenger et al., 2020) and cell membrane (*δ*_*φ*_=0.01 μm) (Alberts et al., 2014) by using the formula for the interface width of the phase-field function (Seirin-Lee et al., 2016) such that:
(12)

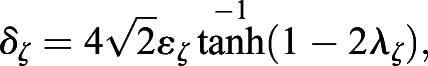
where *ζ*={0, *φ*}, and *λ*_*ζ*_ is the value defining the interface region of the eggshell as 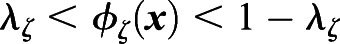. The detailed values of ε_0_ and ε_*φ*_ are given as ε_0_=3.59×10^−2^ and ε_*φ*_=4.86×10^−3^ by choosing *λ*_0_=*λ*_*φ*_=0.27, which defines the regions of the cells as 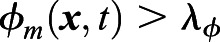. The internal region of the eggshell was defined as 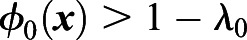, where the region of eggshell thickness was excluded. A schematic of the internal region of the eggshell and cell region is shown in Fig. 1C. ES was calculated by:
(13)

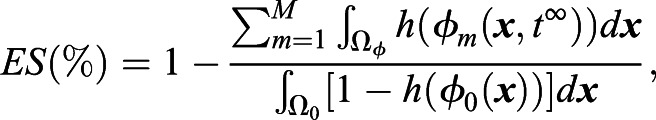
where *t*^∞^ is the time when the cell arrangement approaches a stable state, 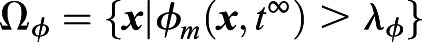 and 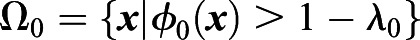.

The spatial size and temporal scale were estimated by comparing the quantitative and qualitative dynamics of the cell arrangement in simulations with live imaging data from the two-cell and four-cell stages of *C. elegans* embryos. From the experimental data, we approximated the interval of the cell division time to 4 min (Table S1). Using these data, we estimated the time scale of the model as *t*=14 s. The area of the system was Ω=95.5 μm×47.25 μm, as estimated by the wild-type *C. elegans* embryo size (long diameter=54 μm and short diameter=29.6 μm) and the non-dimensional numerical space Ω=[0, 1.8]×[0, 0.9]. The cell volumes (*V*_*m*_) were estimated as a relative scale compared with the eggshell size, based on experimental data (Fig. S4).

The other parameter values for the phase-field functions were chosen as *μ*_0_=0.025, *μ*=1.0, *α*_0_=200, *α*_*v*_=300, *β*_0_=50, *β*_*φ*_=30. The strength of the attraction between the cells were chosen as *γ*_24_=*γ*_34_=0.002, *γ*_13_=0.003, *γ*_12_=0.008, *γ*_23_=0.006. The surface tension *κ*_*s*_ varied from 0 to 13. Note that the scales of these kinetic parameters in the phase-field functions are determined depending on the interface values of the phase-field function. Because we estimated the parameters affecting the interface dynamics of the phase-field function (the thickness of eggshell and cell membrane) from experimental data, our simulations can represent the results within a biologically feasible range of parameter values.

### Microscopy observation of *C. elegans* embryos

In Figs 1-3, we used the microscopy images of the *C. elegans* embryos obtained in our previous study (Yamamoto and Kimura, 2017). The methods used to obtain these images were described in this paper. Briefly, phase-contrast images were acquired at room temperature under an inverted microscope (Axiovert 100; Carl Zeiss) equipped with a 40×, 0.70 N.A. objective (Plan-Neofluar; Carl Zeiss) and a CCD camera (ORCA-100; Hamamatsu Photonics). When the cell arrangement was not clear only from the image from a single direction, the embryos were rotated by an eyelash to identify the arrangement. To change the AR of the eggshell, mutants of the *dpy-11* and *lon-1* genes were used in combination with RNAi-mediated gene knockdown of the *C27D9.1* gene. The cell arrangement of the T-reverse type was observed when we knocked down the *hmr-1* and *hmp-2* genes using RNAi, which encodes the proteins responsible for cell adhesion.

### Observation of *Cephalobus* sp. embryos

DWF1301 was obtained from *Caenorhabditis* Genetics Center (CGC) and maintained following a standard *C. elegans* method (Brenner, 1974). To mount the embryo under a microscope, an adult worm was cut open in water and an embryo was mounted on a 1.5% agarose pad, mounted under a coverslip, and sealed with VALAP (Gerholda et al., 2018; Gomez and Schvarzstein, 2018; Jankele et al., 2021). DIC images were acquired at room temperature with an upright microscope (BX51; Olympus) equipped with a 60×, 1.20 N.A. water-immersion objective (UPlanSApo; Olympus) and a CCD camera (ORCA-ER; Hamamatsu Photonics).

### Observation of *perm-1* knockdown *C. elegans* embryos

Knockdown of *perm-1* was performed using the feeding RNAi method (Kamath et al., 2000), by incubating hermaphrodite worms on RNAi plates for 17-23 h at 22°C. To obtain embryos with a variety of ARs, we used CAL0332 (GFP::PH) and CAL2152 (GFP::PH; *lon-1*) strains and feeding RNAi of the *C27D9.1* gene for the latter strain (Yamamoto and Kimura, 2017).

Because the *perm-1* (RNAi) embryos are fragile, we avoided placing the embryos under pressure. For this purpose, we placed the embryos into a spot of 100 mM or 150 mM KCl solution on a coverslip and covered the solution with an oil (Halocarbon oil700, Sigma-Aldrich) to avoid evaporation.

We used a spinning disk confocal system with a 2× intermediate magnification (CSU-MP; Yokogawa) mounted on an inverted microscope (IX71; Olympus) equipped with a 40×1.25 NA, silicone-immersion objective, to characterize the cell arrangement. The specimen was excited with a 488 nm laser with 10% power for 120 ms exposure with confocal sectioning at an interval of 0.5 μm for 61 slices (total 30 μm). Time-lapse imaging was conducted at 1 min intervals. The acquisition system was controlled using the NIS elements software (Nikon). Three-dimensional images were reconstructed using Imaris software (Oxford Instruments). Images were analyzed using ImageJ software (National Institutes of Health). For Fig. 5A,B (fluorescence), the image contrast was adjusted to clarify the borders of the cells.

### Statistical tests

Statistical testing to evaluate the significance of the concentration of a particular group in a population was performed by calculating the combinations. For example, in Fig. 5C, we calculated the probability of the non-diamond arrangements to be concentrated in ES>15% for AR>2.2. In total, we obtained 36 embryos with AR>2.2, and 12 were non-diamond. Among the embryos, 22 embryos in total were ES>15%, and ten were non-diamond. The combination of picking 22 embryos with ES>15% from 36 in total is _36_C_22_. Similarly, the combination of picking ten embryos from 12 non-diamond embryos is _12_C_10_, and 12 out of 24 diamond embryos is _24_C_12_. Therefore, the probability of obtaining the results by chance is 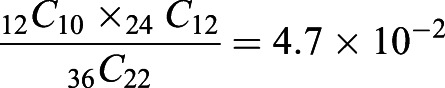.

A binomial test was used to evaluate the significance of the occurrence. For example, in Fig. 5C, we observed nine out of 20 embryos with high ES to show a non-diamond arrangement, whereas in the control, the expectation of non-diamond was 15%. The probability of observing nine or more events out of 20, where the expectation rate is 15%, was calculated from the cumulative binomial distribution.

Calculations were conducted using Microsoft Excel software.

## Supplementary Material

Supplementary information

Reviewer comments

## References

[DEV200401C1] Akiyama, M., Tero, A. and Kobayashi, R. (2010). A mathematical model of cleavage. *J. Theor. Biol.* 264, 84-94. 10.1016/j.jtbi.2009.12.016 20045703

[DEV200401C2] Akiyama, M., Nonomura, M., Tero, A. and Kobayashi, R. (2018). Numerical study on spindle positioning using phase field method. *Phys. Biol.* 16, 016005. 10.1088/1478-3975/aaee45 30485253

[DEV200401C3] Alberts, B., Johnson, A., Lewis, J., Raff, M., Roberts, K. and Walter, P. (2014). *Molecular Biology of the Cell*. New York: Garland Science.

[DEV200401C4] Ansari, T. Q., Huang, H. and Shi, S.-Q. (2021). Phase field modeling for the morphological and microstructural evolution of metallic materials under environmental attack. *Comput. Materials* 7, 143. 10.1038/s41524-021-00612-7

[DEV200401C5] Baena-López, L. A., Baonza, A. and García-Bellido, A. (2005). The orientation of cell divisions determines the shape of *Drosophila* organs. *Curr. Biol.* 15, 1640-1644. 10.1016/j.cub.2005.07.062 16169485

[DEV200401C6] Bowerman, B., Tax, F. E., Thomas, J. H. and Priess, J. R. (1992). Cell interactions involved in development of the bilaterally symmetrical intestinal valve cells during embryogenesis in Caenorhabditis elegans. *Development (Cambridge, England)* 116, 1113-1122. 10.1242/dev.116.4.1113 1295733

[DEV200401C7] Brenner, S. (1974). The genetics of Caenorhabditis elegans. *Genetics* 77, 71-94. 10.1093/genetics/77.1.71 4366476 PMC1213120

[DEV200401C8] Carvalho, A., Olson, S. K., Gutierrez, E., Zhang, K., Noble, L. B., Zanin, E., Desai, A., Groisman, A. and Oegema, K. (2011). Acute drug treatment in the early *C. elegans* embryo. *PloS ONE* 6, e24656. 10.1371/journal.pone.0024656 21935434 PMC3173474

[DEV200401C9] Cheng, N. N., Kirby, C. M. and Kemphues, K. J. (1995). Control of cleavage spindle orientation in *Caenorhubditis elegans*: the role of the genes par-2 and par-3. *Genetics* 139, 549-559. 10.1093/genetics/139.2.549 7713417 PMC1206366

[DEV200401C10] Coffman, V. C., McDermott, M. B. A., Shtyllac, B. and Dawes, A. T. (2016). Stronger net posterior cortical forces and asymmetric microtubule arrays produce simultaneous centration and rotation of the pronuclear complex in the early *Caenorhabditis elegans* embryo. *Mol. Biol. Cell* 27, 3550-3562. 10.1091/mbc.e16-06-0430 27733624 PMC5221587

[DEV200401C11] Dolinski, C., Baldwin, J. G. and Thomas, W. K. (2001). Comparative survey of early embryogenesis of Secernentea (nematoda), with phylogenetic implications. *Can. J. Zool.* 79, 82-94. 10.1139/z00-179

[DEV200401C12] Fickentscher, R., Struntz, P. and Weiss, M. (2013). Mechanical cues in the early embryogenesis of *Caenorhabditis elegans*. *Biophys. J.* 105, 1805-1811. 10.1016/j.bpj.2013.09.005 24138856 PMC3797578

[DEV200401C13] Gerholda, A. R., Pouparta, V., Labbé, J.-C. and Maddox, P. S. (2018). Spindle assembly checkpoint strength is linked to cell fate in the *Caenorhabditis elegans* embryo. *MBoC* 29, 1435-1448. 10.1091/mbc.E18-04-0215 29688794 PMC6014101

[DEV200401C14] Giammona, J. and Campas, O. (2021). Physical constraints on early blastomere packings. *PLoS Comput. Biol.* 17, e1007994. 10.1371/journal.pcbi.1007994 33497383 PMC7864451

[DEV200401C15] Gilbert, S. F. and Michael, J. F. (2019). *Developmental Biology*, 12th edn. Sutherland, MA: Sinauer Associates Inc.

[DEV200401C16] Gloerich, M., Bianchini, J. M., Siemers, K. A., Cohen, D. J. and Nelson, W. J. (2016). Cell division orientation is coupled to cell-cell adhesion by the E-cadherin/LGN complex. *Nat. Commun.* 8, 13996. 10.1038/ncomms13996 PMC5216124 28045117

[DEV200401C17] Gomez, K. A. R. and Schvarzstein, M. (2018). Immobilization nematodes for live imaging using an agarose pad produced with a vinyl record. *Micropubl. Biol.*, 10.17912/QG0J-VT85 10.17912/QG0J-VT85 PMC7282523 32550397

[DEV200401C18] Gönczy, P. (2005). Asymmetric cell division and axis formation in the embryo. WormBook.org. 10.1895/wormbook.1.30.1

[DEV200401C19] Jankele, R., Jelier, R. and Gönczy, P. (2021). Physically asymmetric division of the *C. elegans* zygote ensures invariably successful embryogenesis. *eLife* 10, e61714. 10.7554/eLife.61714 33620314 PMC7972452

[DEV200401C20] Kajita, A. and Yamamura, M. (2002). Physical modeling of the cellular arrangement in *C. elegans* early embryo: Effect of rounding and stiffening of the cells. *Genome Inform.* 13, 224-232. 14571391

[DEV200401C21] Kamath, R. S., Martinez-Campos, M., Zipperlen, P., Fraser, A. G. and Ahringer, J. (2000). Effectiveness of specific RNA-mediated interference through ingested double-stranded RNA in *Caenorhabditis elegans*. *Genome Biol.* 2, research0002.1. 10.1186/gb-2000-2-1-research0002 11178279 PMC17598

[DEV200401C22] Kimura, A. and Onami, S. (2007). Local cortical pulling-force repression switches centrosomal centration and posterior displacement in *C. elegans*. *J. Cell Biol.* 179, 1347-1354. 10.1083/jcb.200706005 18158330 PMC2373484

[DEV200401C23] Krenger, R., Burri, J. T., Lehnert, T., Nelson, B. J. and Gijs, M. A. M. (2020). Force microscopy of the *Caenorhabditis elegans* embryonic eggshell. *Microsyst. Nanoeng.* 6, e29. 10.1038/s41378-020-0137-3 PMC7196560 32382445

[DEV200401C24] Manuel Théry, A. J.-D., Racine, V., Bornens, M. and Julicher, F. (2007). Experimental and theoretical study of mitotic spindle orientation. *Nature* 447, 493-496. 10.1038/nature05786 17495931

[DEV200401C25] Mickey, K. M., Mello, C. C., Montgomery, M. K., Fire, A. and Priess, J. R. (1996). An inductive interaction in 4-cell stage C. elegans embryos involves APX-1 expression in the signalling cell. *Development (Cambridge, England)* 122, 1791-1798. 10.1242/dev.122.6.1791 8674418

[DEV200401C26] Minc, N., Burgess, D. and Chang, F. (2011). Influence of cell geometry on division-plane positioning. *Cell* 144, 414-426. 10.1016/j.cell.2011.01.016 21295701 PMC3048034

[DEV200401C27] Moure, A. and Gomez, H. (2021). Phase-field modeling of individual and collective cell migration. *Arch. Comput. Methods Eng.* 28, 311-344. 10.1007/s11831-019-09377-1

[DEV200401C28] Nonomura, M. (2012). Study on multicellular systems using a phase field model. *PLoS ONE* 7, e33501. 10.1371/journal.pone.0033501 22539943 PMC3335162

[DEV200401C29] Pierre, A., Sallé, J., Wühr, M. and Minc, N. (2016). Generic theoretical models to predict division patterns of cleaving embryos. *Dev. Cell* 39, 667-682. 10.1016/j.devcel.2016.11.018 27997824 PMC5180451

[DEV200401C30] Provatas, N. and Elder, K. (2010). *Phase-Field Methods in Materials Science and Engineering*. Wiley. ISBN:978-3-527-40747-7.

[DEV200401C31] Saito, N. and Sawai, S. (2021). Three-dimensional morphodynamic simulations of macropinocytic cups. *iScience* 24, 1-22. 10.1016/j.isci.2021.103087 PMC8560551 34755081

[DEV200401C32] Schulze, J. and Schierenberg, E. (2011). Evolution of embryonic development in nematodes. *EvoDevo* 2, 18. 10.1186/2041-9139-2-18 21929824 PMC3195109

[DEV200401C33] Seirin-Lee, S. (2016). Lateral inhibition-induced pattern formation controlled by the size and geometry of the cell. *J. Theor. Biol.* 404, 51-65. 10.1016/j.jtbi.2016.05.025 27229622

[DEV200401C34] Seirin-Lee, S., Tashiro, S., Awazu, A. and Kobayashi, R. (2016). A new application of the phase-field method for understanding the reorganization mechanisms of nuclear architecture. *J. Math. Biol.* 74, 333-354. 10.1007/s00285-016-1031-3 27241726 PMC5206286

[DEV200401C35] Seirin-Lee, S., Osakada, F., Takeda, J., Tashiro, S., Kobayashi, R., Yamamoto, T. and Ochiai, H. (2020). Role of dynamic nuclear deformation on genomic architecture reorganization. *PLoS Comput. Biol.* 15, e1007289. 10.1371/journal.pcbi.1007289 PMC6738595 31509522

[DEV200401C36] Shahbazi, M. N. (2020). Mechanisms of human embryo development: from cell fate to tissue shape and back. *Development* 147, dev190629. 10.1242/dev.190629 32680920 PMC7375473

[DEV200401C37] Shao, D., Rappel, W.-J. and Levine, H. (2010). Computational model for cell morphodynamics. *Phys. Rev. Letter* 105, 108104. 10.1103/PhysRevLett.105.108104 PMC3048783 20867552

[DEV200401C38] Shao, D., Levine, H. and Rappel, W.-J. (2012). Coupling actin flow, adhesion, and morphology in a computational cell motility model. *Proc. Natl. Acad. Sci. USA* 109, 6851-6856. 10.1073/pnas.1203252109 22493219 PMC3344950

[DEV200401C39] Takagi, T. and Yamanaka, A. (2012). *Phase-Field Method: PFM*, 1st edn. Yokendo: Tokyo. ISBN:978-4-8425-0492-6.

[DEV200401C40] Taniguchi, D., Ishihara, S., Oonuki, T., Honda-Kitahara, M., Kaneko, K. and Sawai, S. (2013). Phase geometries of two-dimensional excitable waves govern self-organized morphodynamics of amoeboid cells. *Proc. Natl. Acad. Sci. USA* 110, 1218025110. 10.1073/pnas.1218025110 PMC3612638 23479620

[DEV200401C41] Yamamoto, K. and Kimura, A. (2017). An asymmetric attraction model for the diversity and robustness of cell arrangement in nematodes. *Development* 144, 4437-4449. 10.1242/dev.154609 29183946 PMC5769634

