# Supplementary material for: The extra-embryonic space and the local contour are crucial geometric constraints regulating cell arrangement
Source: Development. 2022 May 12;149(9):dev200401. doi: 10.1242/dev.200401 (PMC9148568; doi:10.1242/dev.200401)
Supplement: Supplementary information [file develop-149-200401-s1.pdf]

Supplementary Materials and Methods

Formulation of energy functionals

We designed the model using the energy functions defined by the eggshell, $\phi_0(\mathbf{x}, t)$, and cells, $\phi_m(\mathbf{x}, t)$ $(1 \leq m \leq N)$, where $\mathbf{x} \in \Omega$ in $\mathbf{R}^n$ and $t > 0$. M represents the total number of cells and $M = 4$ in the simulations. For the details of introduction for phase-field modeling, refer to the Appendix of Seirin-Lee et al. (2016), Provatas and Elder (2010), and Takagi and Yamanaka (2012).

First, we defined the basal free energy functions according to the following equation:

$$E_0 = \int_\Omega \left[\frac{\epsilon_0^2}{2} |\nabla \phi_0|^2 + g(\phi_0) \right] d\mathbf{x} + \kappa_s \sum_{m=1}^{M} \int_\Omega \left[\frac{\epsilon_\phi^2}{2} |\nabla \phi_m|^2 + g(\phi_m) \right] d\mathbf{x},$$

Development • Supplementary information

where $\epsilon_\phi, \epsilon_\psi > 0$ are gradient coefficients, κ_s is the surface tension, and $g(\phi) = \phi^2(1-\phi)^2/4$.

Next, we defined the territory (repulsive) conditions between cells as follows.

$$E_1 = \underbrace{\frac{\beta_0}{30}\sum_{m=1}^{M}\int h(\phi_0)h(\phi_m)d\mathbf{x}}_{S_1} + \underbrace{\frac{\beta_\phi}{30}\sum_{m\neq n}^{M}\int h(\phi_n)h(\phi_m)d\mathbf{x}}_{S_2},$$

where β_0 and β_ϕ are positive constants and indicate the intensities of the domain territories. $h(\phi_m)$ is given to $h(\phi_m) = \phi_m^3(10 - 15\phi_m + 6\phi_m^2)$. The term S_1 defines the territory of the eggshell, and S_2 defines the repulsive effect between cells.

In the third step, we defined the eggshell and cell volumes.

$$E_2 = \underbrace{\frac{\alpha_0}{60}\left[\int_\Omega [1 - h(\phi_0)]d\mathbf{x} - V_0\right]^2}_{R_1} + \underbrace{\frac{\alpha_V}{60}\sum_{m=1}^{M}[V_m(t) - \bar{V}_m]^2}_{R_2}$$

where $\alpha_0, \alpha_V > 0$ are the energy intensity constants of each volume. The first term corresponds to the eggshell volume, because $h(\phi_0 = 0) = 0$ and $h(\phi_0 = 1) = 1$. Namely, the the eggshell volume (V_0) is calculated by

$$V_0 \equiv \int_\Omega [1 - h(\phi_0(\mathbf{x}))]d\mathbf{x}.$$

R_1 indicates that the eggshell volume is V_0, and R_2 defines the m-th cell volume as $\bar{V}_m$. The m-th cell volume ($V_m(t)$) is calculated by

$$V_m(t) \equiv \int_\Omega h(\phi_m(\mathbf{x}, t))d\mathbf{x}.$$

In the final step, we formulated cell attraction by

$$E_3 = \sum_{m,i=1(m\neq i)}^{M} \frac{\gamma_{mi}}{2}\int \nabla h(\phi_m)\cdot\nabla h(\phi_i)d\mathbf{x},$$

where $\gamma_{mi} > 0$ is a constant that determines the attraction intensity between the m-th cell and the i-th cell.

The total energy of the cell dynamics is given as

$$E = E_0 + E_1 + E_2 + E_3.$$

With the functional derivatives of the equation above with respect to ϕ_0 and ϕ_m $(1 \leq m \leq M)$, we drove the time for the system to evolve, satisfying

$$\frac{\partial \phi_m}{\partial t} = -\mu \frac{\delta E}{\delta \phi_m} \quad (0 \leq m \leq M)$$

where μ is a positive constant that represents the mobility of each phase-field function.

Development • Supplementary information

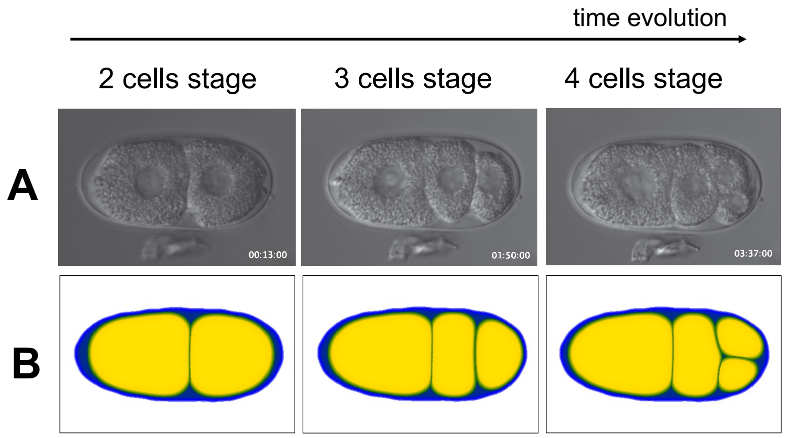

Fig. S1. The T-reverse type arrangement observed in the 4-cell stage of the *Cephalobus sp.* embryo and the reproduction by cell morphology model. (A) DIC images of the early embryogenesis of the *Cephalobus sp.* embryo. Time is indicated in h:min: s from the start of imaging. (B) T-reversed arrangement was successfully reproduced using the cell morphology model. The eggshell was the same as the actual eggshell shape of (A). The cell size, ES ratio, and cell division axis were determined based on the image data of (A).

Table S1. Data for the cell areas and interval of division time. We used 4-cell stage *C. elegans* embryos of the wild-type. Each cell area was calculated as the ratio of the eggshell area. $\bar{x}$ and σ are the average and standard deviations, respectively. The data of 222 embryos were analyzed to determine the cell areas, and the live imaging data of 10 embryos were analyzed for their division times.

**Cell area**	$\bar{x} \pm \sigma$	**Division time**	
ABa cell	26 ±4 (%)	AB cell (t_1)	
ABp cell	21 ±5 (%)	P_1 cell (t_2)	
EMS cell	21 ±5 (%)	$t_2 - t_1$	6±2 (min)
P_2 cell	18 ±4 (%)		

Development • Supplementary information

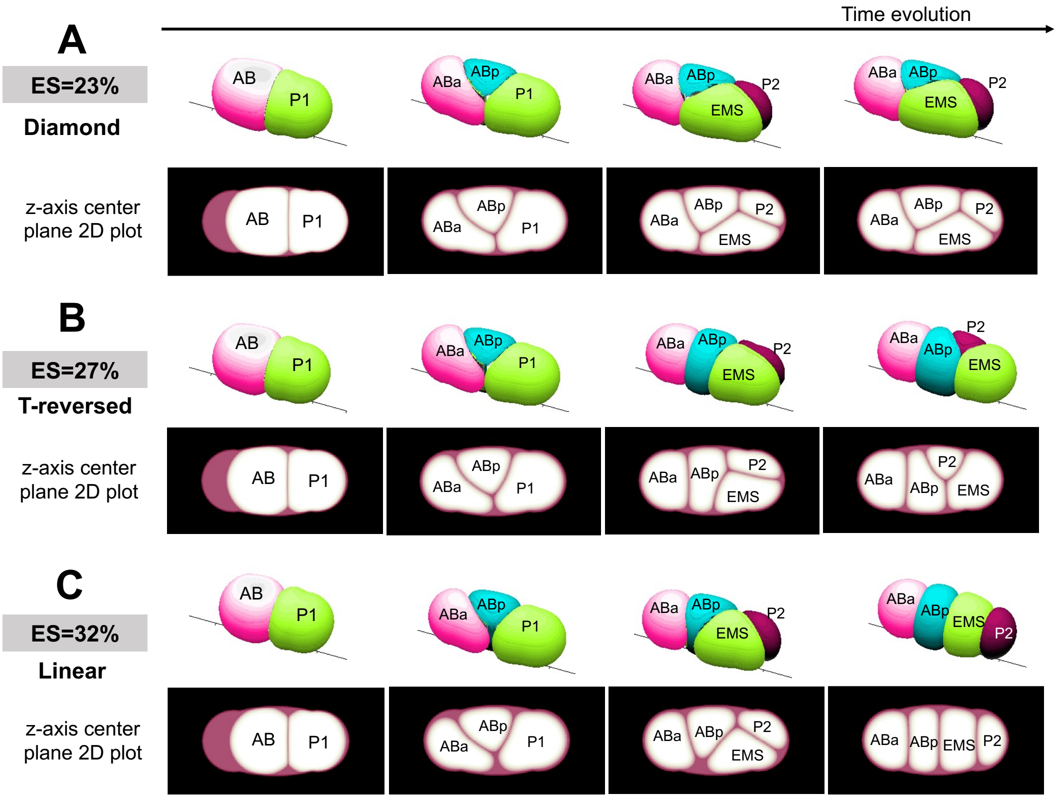

Fig. S2. Diversity of cell arrangements by ES ratio in 3D model. The 3D simulation results by changing the ES ratio = [1 − (Total volume of cells/Internal volume of eggshell)](%). The detailed parameter values are same as in 2D case.

Development • Supplementary information

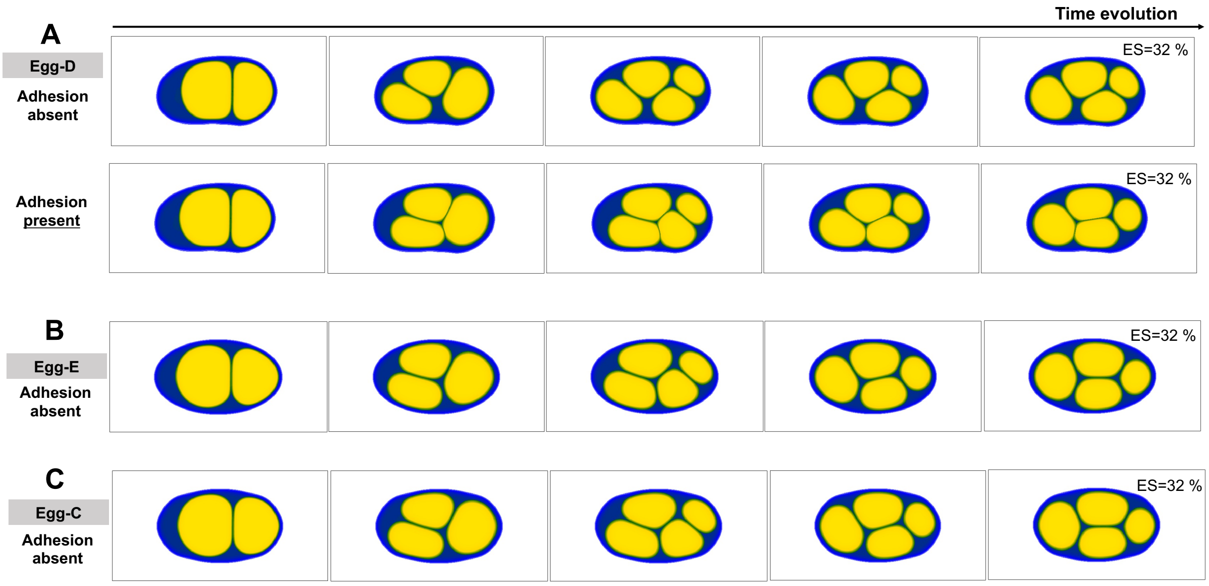

Fig. S3. Examples of the impact of precise eggshell contour. ES ratios of the ex-amples are all 32 %. All the simulation conditions, except for eggshell shape and adhesion absent/present, are the same in (A)-(C). Egg-D eggshell drives the T-reversed arrangement when cell adhesion is absent (A, upper panels). In contrast, Egg-E eggshell (B) and Egg-C (C) drive the diamond arrangement when cell adhesion is absent with the same ES ratio as in the eggshell case. This proves that the precise contour of eggshell leads to different cell arrangement. On the one hand, when cell adhesion is introduced, the robustness of diamond arrangement increases (A, lower panels)

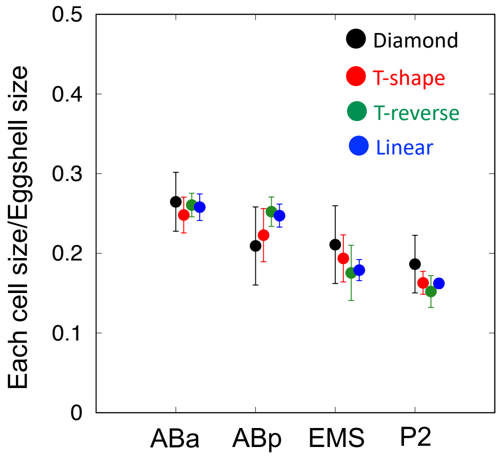

Fig. S4. Cell size of the *C. elegans* embryo in the 4-cell stage. For the relative cell size to eggshell size, data from 222 embryos were analyzed for cell areas. The points indicate the average and the bars indicate the standard deviations.

Development • Supplementary information

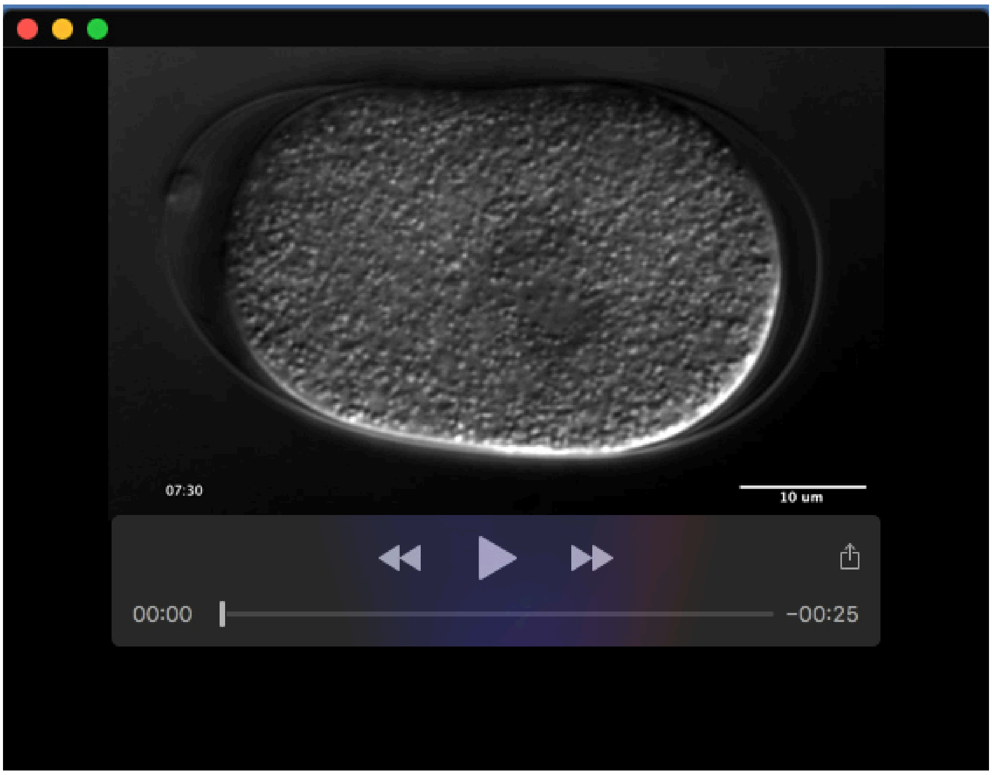

Movie 1. The diamond type arrangement observed in the 4-cell stage of the *C. elegans* embryo. The embryo is identical to that shown in Fig. 2A, and Fig. S1 in Yamamoto and Kimura (2017).

Development • Supplementary information

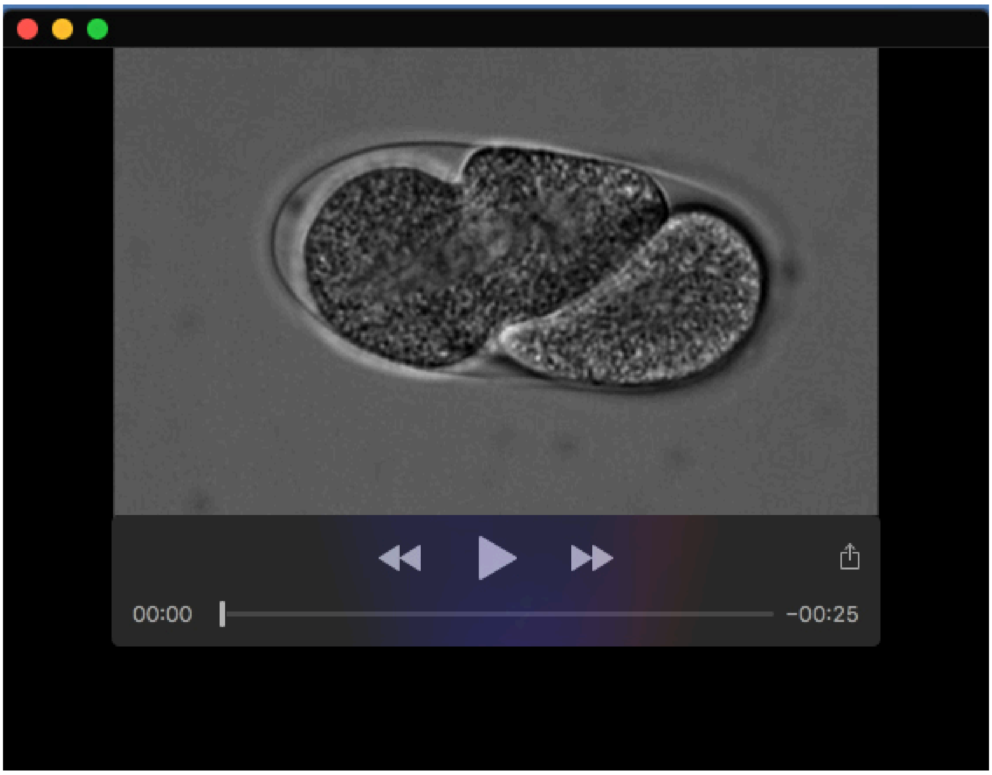

Movie 2. The T-reverse type arrangement observed in the 4-cell stage of the *C. elegans* embryo. The *hmr-1* and *hmp-2* -double-knockdown embryos with a *lon-1(e185)* mutant background. The embryo is identical to that shown in Fig. 1A(a-4) and Fig. 3A of this paper and Fig. 7C of Yamamoto and Kimura (2017).

Development • Supplementary information

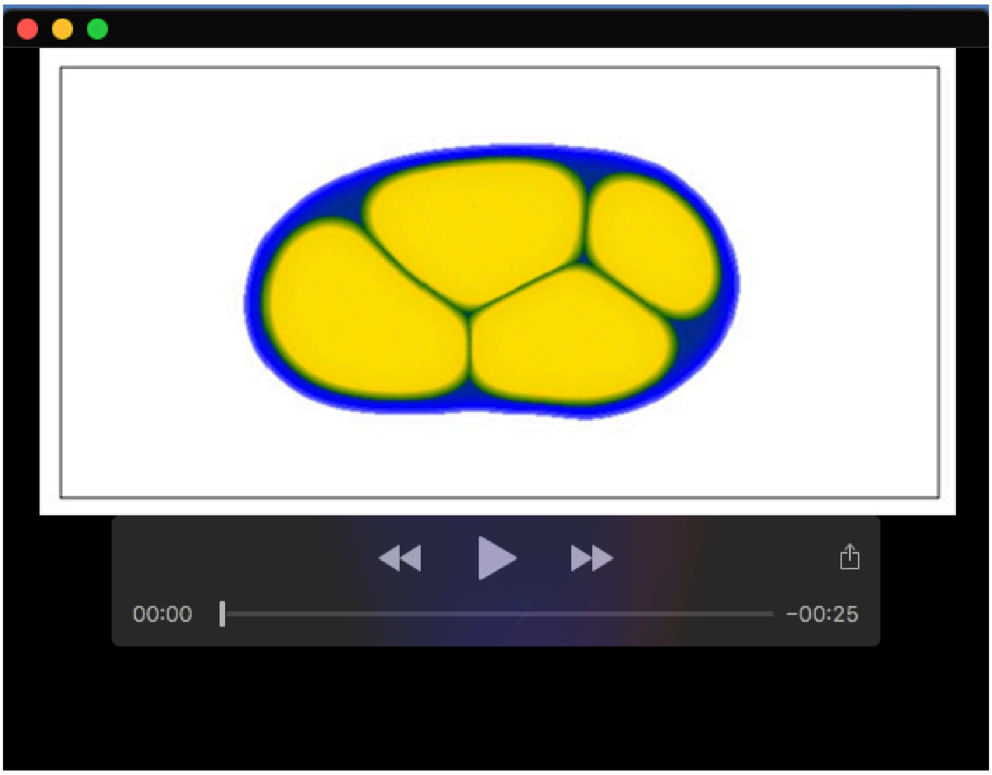

Movie 3. Reproduction of the diamond type arrangement of Movie S1 by the cell morphology model.

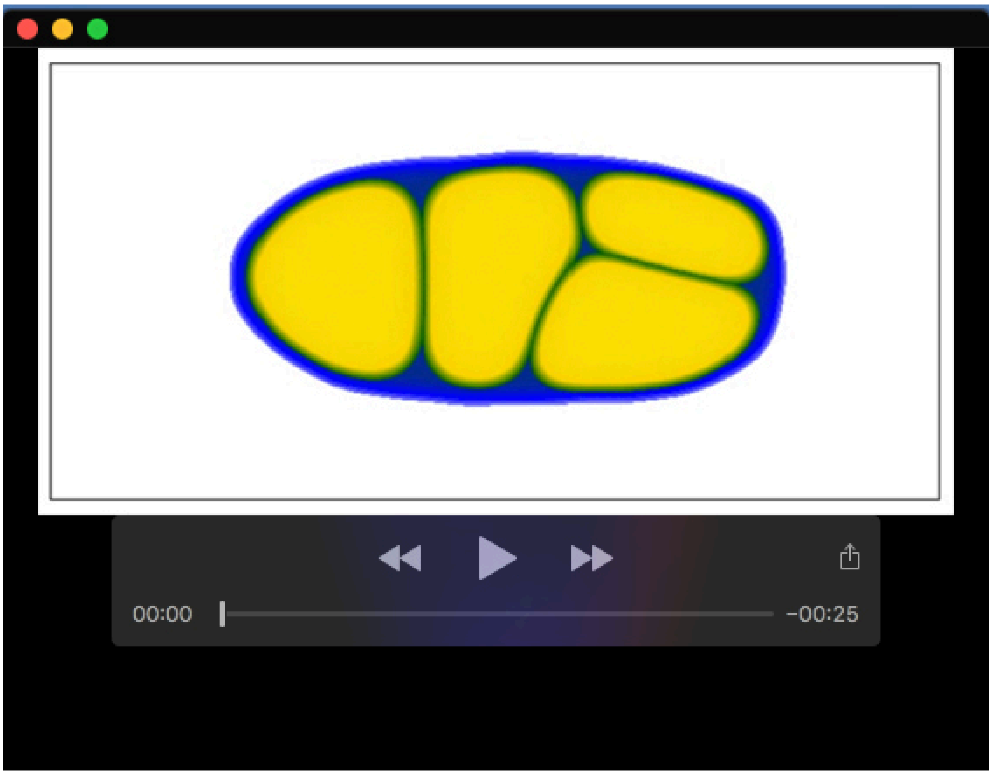

Movie 4. Reproduction of the T-reversed type arrangement of Movie S2 by the cell morphology model.

Development • Supplementary information

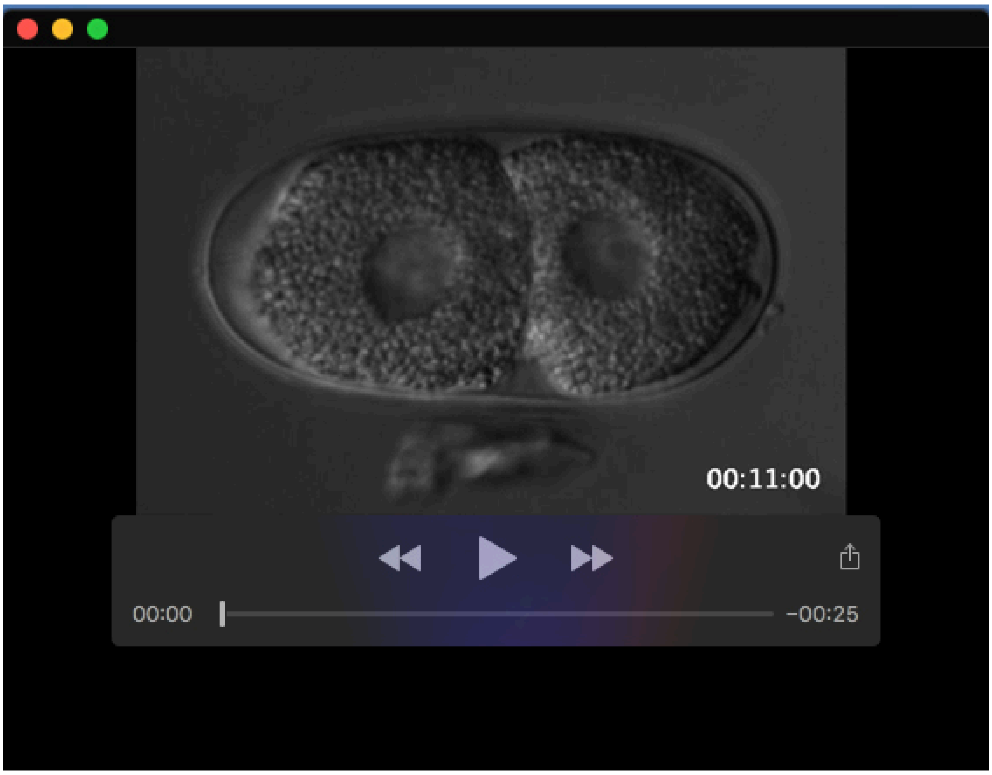

Movie 5. The T-reverse type arrangement observed in the 4-cell stage of the *Cephalobus sp.* embryo. DIC images of the early embryogenesis of the *Cephalobus sp.* embryo. Time is indicated in h:min: s from the start of imaging. The embryo is identical to that shown in Fig. S1.

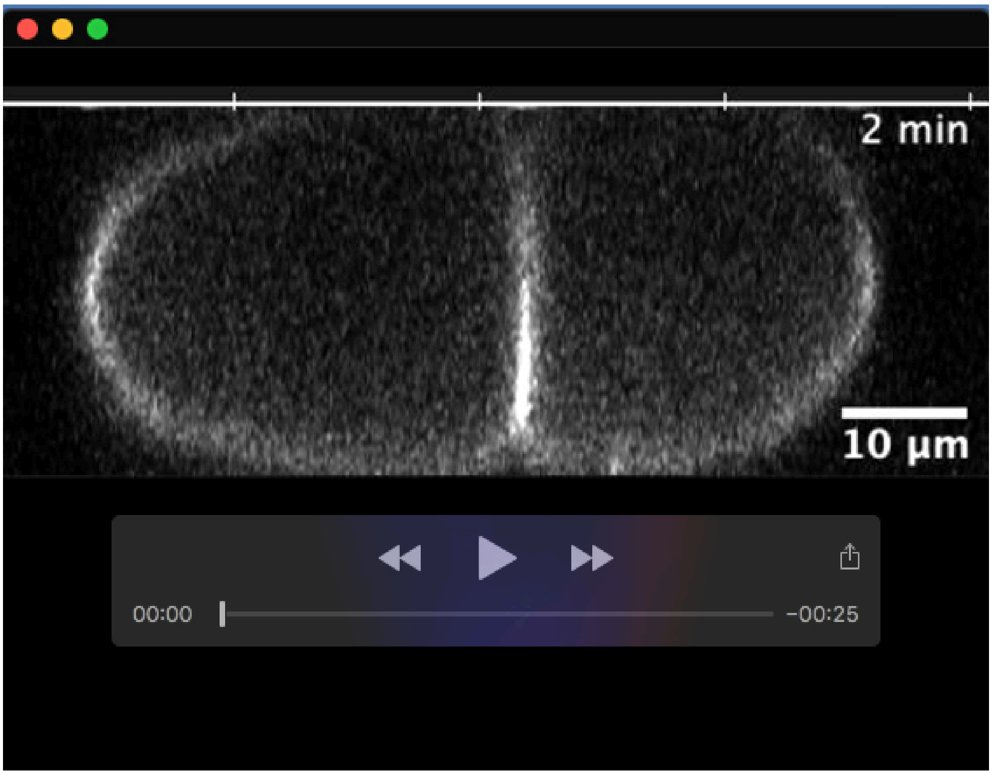

Movie 6. Time lapse images of a representative embryo of *perm-1* (RNAi) in 100 mM KCl solution. The embryo is identical to that shown in Fig. 5A. The elapsed time from the beginning of imaging is indicated in min: s.

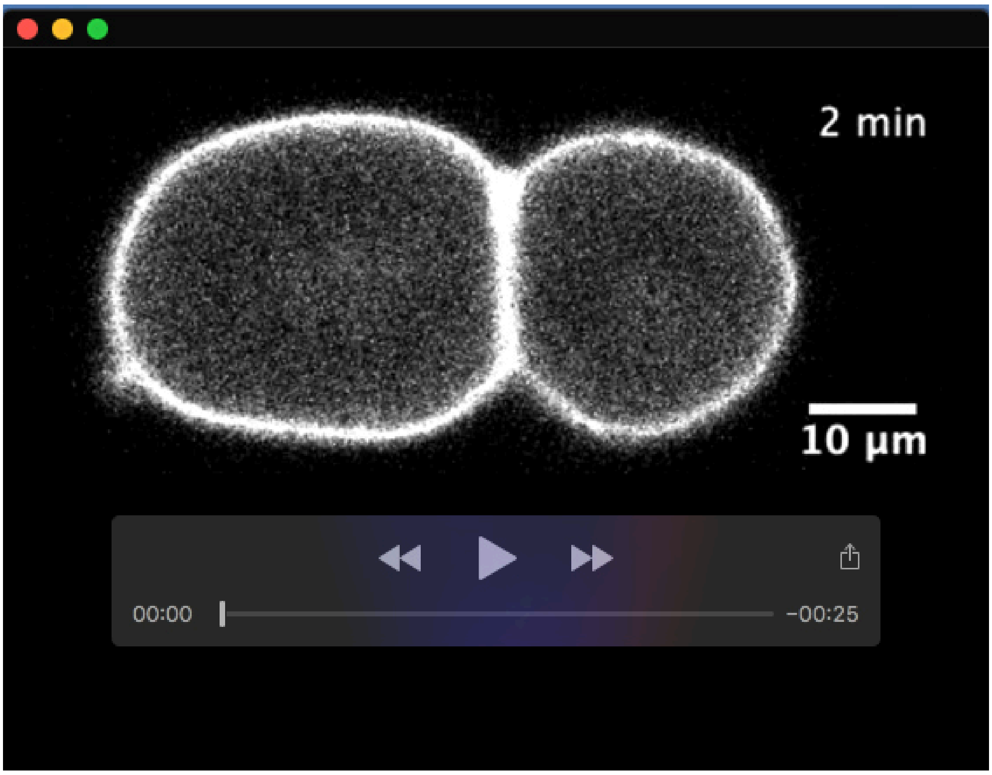

Movie 7. Time lapse images of a representative embryo of *perm-1* (RNAi) in 150 mM KCl solution. The embryo is identical to that shown in Fig. 5B.

References

Provatas, N., Elder, K., 2010. Phase-Field Methods in Materials Science and Engineering. Wiley, ISBN: 978-3-527-40747-7.

Seirin-Lee, S., Tashiro, S., Awazu, A., Kobayashi, R., 2016. A new application of the phase-field method for understanding the reorganization mechanisms of nuclear architecture. Journal of Mathematical Biology 74, 333–354.

Takagi, T., Yamanaka, A., 2012. Phase-field Method : PFM. Yokendo, Tokyo, ISBN: 978-4-8425-0492-6.

Yamamoto, K., Kimura, A., 2017. An asymmetric attraction model for the diversity and robustness of cell arrangement in nematodes. Development 144, 4437–4449.

Development • Supplementary information
