# Supplementary material for: The extra-embryonic space and the local contour are crucial geometric constraints regulating cell arrangement
Source: Development. 2022 May 12;149(9):dev200401. doi: 10.1242/dev.200401 (PMC9148568; doi:10.1242/dev.200401)
Supplement: Reviewer comments [file develop-149-200401_review_history.pdf]

The extra-embryonic space and the local contour are crucial geometric constraints regulating cell arrangement

Sungrim Seirin-Lee, Kazunori Yamamoto and Akatsuki Kimura
DOI: 10.1242/dev.200401

Editor: Thomas Lecuit

Review timeline

First submission received:	13 June 2021
Editorial decision:	27 July 2021
Resubmission:	26 November 2021
Editorial decision:	25 January 2022
First revision received:	10 February 2022
Editorial decision:	9 March 2022
Second revision received:	17 March 2022
Accepted:	23 March 2022

Original submission decision letter

MS ID#: DEVELOP/2021/199899
MS TITLE: The extra-embryonic space is a geometric constraint regulating cell arrangement in nematodes
AUTHORS: Sungrim Seirin-Lee and Akatsuki Kimura
ARTICLE TYPE: Research Article

I have now received all the referees reports on the above manuscript, and have reached a decision. The referees' comments are appended below, or you can access them online: please go to BenchPress and click on the 'Manuscripts with Decisions' queue in the Author Area.

As you will see from their reports, the referees recognise the potential of your work, but they also raise significant concerns about it. Given the nature of these concerns, I am afraid I have little choice other than to reject the paper at this stage.

However, having evaluated the paper, I do recognise the potential importance of this work. I would therefore be prepared to consider as a new submission an extension of this study that contains new experiments, data and discussions and that address fully the major concerns of the referees. In particular, two major points need to be addressed for the manuscript to be considered following revision, though all other comments should be considered as well. First, the paper should not simply account for packing of a mutant (ie. non physiological) condition but also of the wild type (Reviewer 1). Second, as pointed out by the two other reviewers, the role of the extra-embryonic space (the fluid) versus the egg shell per see needs to be distinguisehd and tested as it is not clear at present what provides geometric constraint. The work required goes beyond a standard revision of the paper. Please bear in mind that the referees (who may be different from the present reviewers) will assess the novelty of your work in the context of all previous publications, including those published between now and the time of resubmission.

© 2022. Published by The Company of Biologists under the terms of the Creative Commons Attribution License (https://creativecommons.org/licenses/by/4.0/).

Reviewer 1

Advance Summary and Potential Significance to Field

This manuscript reports modeling and experiments to address how cells become arranged in a confined space, using the C. elegans 4-cell embryo as a model. The authors report that one cell arrangement that they had observed before after reducing cell adhesion could not be explained by their previous model. The current paper sought to explain this adhesion-deficient arrangement and to understand why arrangements are not fully explained by an embryo's aspect ratio. The authors re-examined images from the earlier paper, and they built a new model that could incorporate more precise variations of eggshell shape. In their model, the more accurate eggshell shapes could recapitulate the unexplained result above from adhesion-deficient embryos. Next, they explored the role of extra space inside the eggshell. Simulations showed that extra space could affect cell arrangement, and correlative results of variation in real embryos extra-embryonic space matched this. The roles of adhesion, aspect ratio, and extra space were examined by modeling in combinations. Lastly, the position of the extra space was shown in the model to matter, and again, correlative data from embryos matched this.

Comments for the author

The Kimura lab has a strong history of modeling and experiments that have made significant, novel contributions to understanding developmental mechanisms, in line with the central publication criterion of Development. However, I am not convinced that the current manuscript does this. Much of this manuscript aims to explain an arrangement of cells that does not occur in normal embryos or aims to demonstrate something that is already known: the space available to cells, together with cell adhesion, affects cells' final positions.

Minor comment:
The conclusions that are based on the empirical data can't be assessed without statistical tests, which I could not find.

Reviewer 2

Advance Summary and Potential Significance to Field

Background and summary
In this manuscript the authors report that the extra-embryonic space in C. elegans embryos provides a geometric constraint that is instrumental for the arrangements of embryonic cells in 2-4 cell embryos. This work builds on their earlier work published in Development (Yamamoto and Kimura, 2017) in which the authors showed that egg shell shape is a determining factor for cell arrangements. In this earlier study they imaged embryos with varying egg shell geometries, and further refined an existing theoretical model from the Weiss lab (Fickentscher et al 2013). Although this refinement improved the predictive power of the model, it could not recapitulate two experimentally observed features: 1) a particular cell arrangement, termed T-reverse, observed in one genetic background, and 2) embryos with similar shapes (i.e. aspect ratios) can display different arrangements. In this manuscript the authors developed a new theoretical description based on multi-phase field methodology. Moreover, rather than assuming a perfect ellipsoidal egg shell shape (as was done in the earlier work), the experimentally measured egg shell shape was used as input for the theoretical model. This model could reproduce the experimentally observed arrangements and showed that the precise egg shell shape is an important factor for determining cell arrangements. I appreciate the theoretical endeavour, and think the results are interesting. I do however have several major criticisms listed below.

Comments for the author

Major criticisms
1) In their previous work, as well as in other previous studies, the authors showed that egg shell shape is instrumental for proper positioning of embryonic cells. In this manuscript, the authors propose that the extra-embryonic space is instrumental. However, I do not follow this logic and I

© 2022. Published by The Company of Biologists under the terms of the Creative Commons Attribution License (https://creativecommons.org/licenses/by/4.0/).

think that their results point again to the egg shell providing the geometric constraints instead. These egg shell-derived geometric constraints determine how cells arrange themselves, and in turn, the shape and position of the extra embryonic space simply result from cellular arrangements. Moreover, because both the location and shape/arrangement of the extra embryonic space change over developmental time, I don't think calling it a geometric constraint is accurate.

2) The new model that the authors propose is based on the multi-phase field method that describes the time evolution of multiple object boundaries in response to forces. This is different from their previous model that describes cells as soft interacting spheres. Moreover, in the simulation using their new model, the authors used the experimentally measured egg shell shape, rather than assuming a perfect ellipsoid shape, which was done in their previous work. Because their earlier AA model already predicted a range of cellular rearrangements successfully, I wonder whether their earlier model could have worked equally well (or better) if the experimentally measured egg shell geometries were used. In other words, are the authors sure that their new theoretical description is better than their earlier one?

3) The new theoretical model, as it is used here, is 2D. The authors motivate this decision by saying that the cellular arrangement is planar. I can follow this logic, but I am still worried that this 2D model is not physiologically relevant. In the discussion the authors mention that it can be easily extended to a 3D model. I understand that computational costs are limiting a further extensive analysis, but I think the story would be much more convincing if a couple of key conditions could be verified by using the same model in 3D.

4) The authors use the imaging data from their previous paper (Yamamoto & Kimura 2017) to classify different cellular arrangements. This data set was acquired using phase contrast imaging, in which there is no 3D information. Given that the authors performed imaging on embryos uncompressed in the z direction (as showed in Yamamoto & Kimura 2017), a substantial number of embryos classified as linear, might simply be tilted T-shapes viewed down the dorsoventral axis. The authors should rule this out as it may skew the results and affect conclusions.

5) In line 86 the authors mention the multi-phase field method for the first time in the results section. The authors should briefly explain what this method is, what are the differences with earlier models, what are the advantages, and why it is used here. I think that one cannot expect a developmental biology audience to understand it in its current form. Similarly, the rather complex equations 1 and 2 enter the text very abruptly and I feel that they are not explained well. Finally, several variables/constants have not been specified. Altogether, this can be improved and tailored better to a non-physics audience.

6) It is stated in the materials & methods that '…we fixed the direction and position of the cell division plane based on the image of the wild type C. elegans embryo. As geometric constraints have profound effects on cell division orientation in various different organisms/contexts, the authors should verify that it is indeed valid to assume a wild type cell division orientation in the various conditions used.

Minor criticisms
- I would appreciate it if the authors could discuss their work in relation to the preprint from the Zhang lab (Kuang et al, bioRxiv, 2020), that also used phase field modelling to describe cellular arrangements in early C. elegans embryos.

- Numbers indicating cells in the P lineage should be subscript.

- Typo line 45: 'phase-filled'

<u>Reviewer 3</u>

Advance Summary and Potential Significance to Field

In this work, the authors use a phase field model to explore the types and variability of cell packings at the 4 cell stage of C Elegans. The paper makes good use of both simulations and experiments to constraint some geometrical parameters and see the influence of the packing of

© 2022. Published by The Company of Biologists under the terms of the Creative Commons Attribution License (https://creativecommons.org/licenses/by/4.0/).

cells, which is commendable. In particular they propose that the exact shape of the eggshell and the fraction of extra-embryonic space are important predictors for the cell packings - this is an interesting idea supported by both model and data (although see below some questions on it). Despite these strengths, I had two concerns on the manuscript: the first more minor one is that it's quite hard to read (see below) as it jumps back and forth between theory and data, and between past and previous works of the authors, so it would need restructuring. The second more important one is that I'm not sure I understand the argument that the location of the fluid determines the packing (it would seem to me as inputting in the simulations the former to get the latter is a bit of a circular argument, and it would be important to consider other sources of variability that might explain both in a more causal manner, for instance variability in cell tensions/adhesion).

Comments for the author

Major questions:

Existence of other sources of stochasticity/variability, and problem of cause vs effect on the location of fluid (ES) .

The authors write "The coexistence of different types of cell arrangement within the same AR indicates that other aspects of geometrical constraints other than AR play important roles." But I'm not sure i fully follow this argument. Can't this be due to stochasticity in cell processes, rather than geometrical constraints? The authors subsequently show that fluid fraction is a key determinant, as is the exact shape of the boundary, which is interesting, but doesn't exclude other sources of variability - especially to explain the positioning of the fluid. .

Indeed, in Fig. 6, the authors write that " diversity in location of the ES can explain the experimental result with the co-existence of the T-reverse and T-shaped arrangements in similar AR " This was the part of the work I was less sure about. Isn't there to some degree a circular argument going on here? When the authors talk about the relative amount of fluid, and how it changes the packing, this is a highly non-trivial prediction, because it's not clear a priori how the two relate to each other… However, when looking at T-shapes or reverse T-shapes from the first figures, it's already clear that the fluid is located in different regions… but isn't that just a natural - and in fact inevitable consequence of the packing? It seemed to me, at least at first sight, that actively positioning the ES region in the simulation was close to inputting the results that the authors look for… In fact the manuscript finishes by the observation that "The results showed that the T-reversed case has more ES in the anterior side, whereas the T-shaped case has more ES in the posterior side", but as i mentioned this is something that is quite intuitive/visual from Fig. 1A.

The authors mention in Materials and Methods that "As there are no data for the attraction between ABp cells and P2 cells (?34), we used a similar scale for the attraction between the EMS cell and P2 cell (?24)."… but isn't for instance variability on this parameter a strong candidate to explain the variability in the packings and the positioning of the fluid? This would seem at least superficially as a more direct causal mechanism?

Fig. 4 on the correlation between the type of packing and ES fraction:

I see the effect that the authors talk about, but given the amount of noise, is it significant? Furthermore, could the authors plot this in a slightly more "raw" manner, as looking at categories/bins can be a bit difficult to read… For instance, could they make a plot exactly like 4C, but where each point is color-coded in red or black if it's a T-shape or a diamond? This would enable the readers to really see the full raw data and assess the dispersion.

Clarity of the presentation:

In general, the paper is quite hard to read and follow, because it goes back and forth between different types of theoretical investigates and data, but also back and forth between previous and current work from the authors.

In particular, the authors don't explain enough the AA model (past paper), and its key differences with the current one. They keep referring to diverse results of their 2017 paper on this throughout

© 2022. Published by The Company of Biologists under the terms of the Creative Commons Attribution License (https://creativecommons.org/licenses/by/4.0/).

the text, but this becomes a bit unclear. I think the authors should try to make this paper more self-contained. In particular, around page 7-8, they say that both models give different predictions on the T-reverse arrangement, and directly associate with the detailed shape of the egg… but given that the two models are quite different, isn't there a lot other possibilities at this stage? Investigating the AA model with the right shape for instance would seem like the right control?

The theory also seems quite dispersed in different figures, while compacting the experimental observations first might be better. For instance, Fig. 3 doesn't seem very necessary as the key information/model it contains seems to have been looked at much more clearly and systematically in Figure 5? (but are separated by a Fig. 4 of data)

Minor comments:

Recent references such as Kuang et al, BioRxiv or Giammona et al, Plos Comp Biol 2021 also investigate this question of C elegans packing and seem relevant here.

Typos:
line 109: "regenerated actual eggshell shapes" -> i'm not sure what regenerated means in this context
line 186: "can cause variety " -> "can cause variability?/variations?"

Author response to reviewers' comments

Reviewer #1

Reviewer 1 Advance Summary and Potential Significance to Field:
This manuscript reports modeling and experiments to address how cells become arranged in a confined space, using the C. elegans 4-cell embryo as a model. The authors report that one cell arrangement that they had observed before after reducing cell adhesion could not be explained by their previous model. The current paper sought to explain this adhesion-deficient arrangement and to understand why arrangements are not fully explained by an embryo's aspect ratio. The authors re-examined images from the earlier paper, and they built a new model that could incorporate more precise variations of eggshell shape. In their model, the more accurate eggshell shapes could recapitulate the unexplained result above from adhesion-deficient embryos. Next, they explored the role of extra space inside the eggshell. Simulations showed that extra space could affect cell arrangement, and correlative results of variation in real embryos extra-embryonic space matched this. The roles of adhesion, aspect ratio, and extra space were examined by modeling in combinations. Lastly, the position of the extra space was shown in the model to matter, and again, correlative data from embryos matched this.

Comment #1-1
Reviewer 1 Comments for the Author:
The Kimura lab has a strong history of modeling and experiments that have made significant, novel contributions to understanding developmental mechanisms, in line with the central publication criterion of Development. However, I am not convinced that the current manuscript does this. Much of this manuscript aims to explain an arrangement of cells that does not occur in normal embryos or aims to demonstrate something that is already known: the space available to cells, together with cell adhesion, affects cells' final positions.

We thank the reviewer for a positive evaluation of our previous works, and critical comments for the novelty of this work.
To overcome the first part of the comment, we add an experimental observation of another nematode species, Cephalobus sp., whose wildtype shows the T-reverse type arrangement (Fig. S1). The 4-cell stage embryo of Cephalobus sp. showed the T-reverse type arrangement, and our model reproduced the arrangement when we applied the eggshell shape, each cell size, ES ratio, and cell division orientation of the of the Cephalobus sp. embryo (Fig. S1). The new result indicates that our study, which reproduced the T-reverse type arrangement of a mutant of C. elegans embryo for the

© 2022. Published by The Company of Biologists under the terms of the Creative Commons Attribution License (https://creativecommons.org/licenses/by/4.0/).

first time (Movie S5), is also a novel step forward to understand the mechanics underlying the normal (wild-type) embryo of Cephalobus sp. Cephalobus sp. is not only species that shows the T-reverse type arrangement (Dolinski et al. 2001). Therefore, our study should be important to understand the diversity of the development observed in nature.
As for the latter part of the comment, we would like to emphasize that our present work is novel in two ways. First, we showed the importance of local contour for the first time. A subtle change in the shape of eggshell space (e.g. ellipsoid vs capsule) affected cell arrangements. Second, we showed that even a small change in the space available (ES) to cells is critical for the cell arrangement. In a qualitative sense, it is not surprising (as the reviewer pointed out) that the available space affects the cell arrangement. For example, in an extreme situation, we know that the cell arrangement changes if there is no geometrical constrains (e.g. removing eggshell [Edgar et al, Development 120, 443-451, 1994]). However, there have been no studies to date that have examined the effect of precise eggshell geometry (local contour, Fig. 3) and proved quantitatively how the variation of the available space (ES) results in the diversity of cell arrangements, based on both experiment and mathematical approaches (Figs. 4 and 5). There is a full theoretical study that tested the change in the amount of the eggshell space [Giammona & Camp s, PLoS Comp Biol 17, e1007994, 2021], but the study is based on an artificial ellipsoidal eggshell shape and the degree of change in space is large. Moreover, the study is not directly connected to experimental observations. No study had conducted integrated analyses of theory and experiments to examined how the amount and shape of the space affects the cell arrangement prior to the present study. Therefore, we believe our study is the first attempt and will be a future basis of quantitative study that raises the importance of the relationship between the extra-embryonic space and the cell arrangement.
Finally, to further strengthen the novelty of this study, we newly conducted experiments to control the amount of the empty space (Fig. 5 and Result Section 2.6). We were able to prove that the subtle change in the amount of the empty space actually changed the cell arrangement as predicted by our model.

Comment #1-2
Minor comment:
The conclusions that are based on the empirical data can't be assessed without statistical tests, which I could not find.

We conducted a statistical test to show the increase in non-diamond type arrangements with the increase in the empty space (Fig. 5C, Section 2.6: P15: L.226–239, and Fig. 7B, Section 2.8: P20: L.311–315). The way we conducted the tests is described in Materials and Methods (Section 4.7: P.27: L.504–518). Our current simulation is a deterministic model and thus we do not think we need statistical tests for the other parts in the current manuscript.

Reviewer #2

Reviewer 2 Advance Summary and Potential Significance to Field:
Background and summary
In this manuscript the authors report that the extra-embryonic space in C. elegans embryos provides a geometric constraint that is instrumental for the arrangements of embryonic cells in 2-4 cell embryos. This work builds on their earlier work published in Development (Yamamoto and Kimura, 2017) in which the authors showed that egg shell shape is a determining factor for cell arrangements. In this earlier study they imaged embryos with varying egg shell geometries, and further refined an existing theoretical model from the Weiss lab (Fickentscher et al 2013). Although this refinement improved the predictive power of the model, it could not recapitulate two experimentally observed features: 1) a particular cell arrangement, termed T-reverse, observed in one genetic background, and 2) embryos with similar shapes (i.e. aspect ratios) can display different arrangements. In this manuscript the authors developed a new theoretical description based on multi-phase field methodology. Moreover, rather than assuming a perfect ellipsoidal eggshell shape (as was done in the earlier work), the experimentally measured egg shell shape was used as input for the theoretical model. This model could reproduce the experimentally observed arrangements and showed that the precise eggshell shape is an important factor for determining cell arrangements. I appreciate the theoretical endeavour, and think the results are interesting. I do however have several major criticisms listed below.

© 2022. Published by The Company of Biologists under the terms of the Creative Commons Attribution License (https://creativecommons.org/licenses/by/4.0/).

Comment #2-1
Reviewer 2 Comments for the Author:
Major criticisms
1) In their previous work, as well as in other previous studies, the authors showed that eggshell shape is instrumental for proper positioning of embryonic cells. In this manuscript, the authors propose that the extra-embryonic space is instrumental. However, I do not follow this logic and I think that their results point again to the eggshell providing the geometric constraints instead. These eggshell-derived geometric constraints determine how cells arrange themselves, and in turn, the shape and position of the extra embryonic space simply result from cellular arrangements. Moreover, because both the location and shape/arrangement of the extra embryonic space change over developmental time, I don't think calling it a geometric constraint is accurate.

We understand the reviewer's comment and would like to clarify our novelty as follows. We agree with the reviewer that eggshell shape is instrumental in our present study. The novelty of our study is that we demonstrated the importance of (i) the precise shape of the eggshell (i.e. "local contour"), and the approximation to ellipsoids is not enough. The second point is that (ii) the amount of the extra-embryonic space, which is defined by the internal volume of the eggshell and the cells, is also critical for the cell arrangement. To strengthen the latter point, we succeeded to experimentally demonstrate that a subtle change in the amount of the extra-embryonic space indeed changed the cell arrangement. We added the new experimental results to the revised manuscript (Fig. 5 and Result Section 2.6).
Concerned to (iii) the location of the extra-embryonic space, we also agree with the reviewer that the location is a consequence of the cells' positioning, and it is not appropriate to call it a geometric constraint. To focus on the genuine geometric constraints in this study, we decided to remove the effect of the location in the revised manuscript. We believe the removal of this part made the manuscript more self-consistent. We believe the novelty of this study is high enough as we discussed in the previous paragraph.

Comment #2-2
2) The new model that the authors propose is based on the multi-phase field method that describes the time evolution of multiple object boundaries in response to forces. This is different from their previous model that describes cells as soft interacting spheres. Moreover, in the simulation using their new model, the authors used the experimentally measured eggshell shape, rather than assuming a perfect ellipsoid shape, which was done in their previous work. Because their earlier AA model already predicted a range of cellular rearrangements successfully, I wonder whether their earlier model could have worked equally well (or better) if the experimentally measured eggshell geometries were used. In other words, are the authors sure that their new theoretical description is better than their earlier one?

Yes, our new theoretical description is better than our earlier one, in terms of the ease in incorporating precise shape of the eggshell. Our previous model assumed ellipsoidal shapes, and could not incorporate other shapes. (It might be not impossible, but the coding will be very laborious.)
The basic driving force for the cells' movement is similar in both models. They consist of repulsion and attraction between the cells and between the cell and the eggshell. While we did not emphasize much in the manuscript, our new model realizes the repulsion and attraction with surface tension and adhesion of the surfaces, whereas our earlier one realized with imaginary forces acting between the cell centers depending on their distance.
Furthermore, our new model using phase-field method gives a more accurate calculation of ES because each cell geometry is precisely reflected.
We think our current model is better in terms of the ease to correlate the parameters in the model and those in the cell.

Comment #2-3
3) The new theoretical model, as it is used here, is 2D. The authors motivate this decision by saying that the cellular arrangement is planar. I can follow this logic, but I am still worried that this 2D model is not physiologically relevant. In the discussion the authors mention that it can be easily extended to a 3D model. I understand that computational costs are limiting a further extensive analysis, but I think the story would be much more convincing if a couple of key conditions could be verified by using the same model in 3D.

© 2022. Published by The Company of Biologists under the terms of the Creative Commons Attribution License (https://creativecommons.org/licenses/by/4.0/).

We successfully conducted 3D simulations and added in the revised manuscript (Fig. 1F and Fig.S2). The results of 3D simulations were consistent with the 2D simulations. We are happy that our work became more convincing and would like to thank the reviewer for pointing out.

Comment #2-4
4) The authors use the imaging data from their previous paper (Yamamoto & Kimura 2017) to classify different cellular arrangements. This data set was acquired using phase contrast imaging, in which there is no 3D information. Given that the authors performed imaging on embryos uncompressed in the z direction (as showed in Yamamoto & Kimura 2017), a substantial number of embryos classified as linear, might simply be tilted T-shapes viewed down the dorsoventral axis. The authors should rule this out as it may skew the results and affect conclusions.

For the results in the previous paper (Yamamoto & Kimura, 2017), the authors rotated the embryos with eyelash when the arrangement is not clear (e.g. linear or T-shape). Therefore, the mis-classification should be minimum. We added this explanation in the resubmitted manuscript (P. 26, L. 471–472).
For the new results in this paper (Fig. 5), we conducted confocal microscopy with z-sectioning using a membrane marker to avoid the mis-classification.

Comment #2-5
5) In line 86 the authors mention the multi-phase field method for the first time in the results section. The authors should briefly explain what this method is, what are the differences with earlier models, what are the advantages, and why it is used here. I think that one cannot expect a developmental biology audience to understand it in its current form. Similarly, the rather complex equations 1 and 2 enter the text very abruptly and I feel that they are not explained well. Finally, several variables/constants have not been specified. Altogether, this can be improved and tailored better to a non-physics audience.

In the present manuscript, we addressed these points. First, we explained the differences between the current 'cell morphology model' with the earlier 'anisotropic attraction model' (P. 3, L. 36–47; P.4, L.67–78; P.9, L.126–129; P.10, L.146–151). Second, we added explanation for the model for an intuitive understanding of the model (P.4–6). The revisions made clear what the variables/constants are. The values of the variables/constants are specified in Materials and Methods (P.22-23) and SI (P.33-34; Table S1).

Comment #2-6
6) It is stated in the materials & methods that '…we fixed the direction and position of the cell division plane based on the image of the wild type C. elegans embryo. As geometric constraints have profound effects on cell division orientation in various different organisms/contexts, the authors should verify that it is indeed valid to assume a wild type cell division orientation in the various conditions used.

For the lon-1(e185) mutant, C27D9.1 (RNAi), hmr-1 (RNAi), and hmp-2 (RNAi) embryos (Fig. 1B), whose measurements were originally conducted in our previous publication, we had experimentally confirmed that they did not affect the cell division orientation (Yamamoto & Kimura, 2017). For the perm-1 (RNAi), they show abnormalities in minor cases (n=4/62), and these cases were excluded from the further analyses. Therefore, we had verified that we can assume constant orientation of cell divisions in our model. This point is explained in the present manuscript (P.24, L.419–424).

Comment #2-7
Minor criticisms
- I would appreciate it if the authors could discuss their work in relation to the preprint from the Zhang lab (Kuang et al, bioRxiv, 2020), that also used phase field modelling to describe cellular arrangements in early C. elegans embryos.
We added this reference (P.21, L. 341). Thank you for the suggestion.

- Numbers indicating cells in the P lineage should be subscript.

We corrected this point. Thank you for pointing out.
- Typo line 45: 'phase-filled'

© 2022. Published by The Company of Biologists under the terms of the Creative Commons Attribution License (https://creativecommons.org/licenses/by/4.0/).

We corrected this point. Thank you for pointing out.

Reviewer #3
Reviewer 3 Advance Summary and Potential Significance to Field:
In this work, the authors use a phase field model to explore the types and variability of cell packings at the 4 cell stage of C Elegans. The paper makes good use of both simulations and experiments to constraint some geometrical parameters and see the influence of the packing of cells, which is commendable. In particular they propose that the exact shape of the eggshell and the fraction of extra-embryonic space are important predictors for the cell packings - this is an interesting idea supported by both model and data (although see below some questions on it). Despite these strengths, I had two concerns on the manuscript: the first more minor one is that it's quite hard to read (see below) as it jumps back and forth between theory and data, and between past and previous works of the authors, so it would need restructuring. The second more important one is that I'm not sure I understand the argument that the location of the fluid determines the packing (it would seem to me as inputting in the simulations the former to get the latter is a bit of a circular argument, and it would be important to consider other sources of variability that might explain both in a more causal manner, for instance variability in cell tensions/adhesion).

Comment #3-1
Reviewer 3 Comments for the Author:
Major questions:
Existence of other sources of stochasticity/variability, and problem of cause vs effect on the location of fluid (ES) .
The authors write "The coexistence of different types of cell arrangement within the same AR indicates that other aspects of geometrical constraints other than AR play important roles." But I'm not sure i fully follow this argument. Can't this be due to stochasticity in cell processes, rather than geometrical constraints?
The authors subsequently show that fluid fraction is a key determinant, as is the exact shape of the boundary, which is interesting, but doesn't exclude other sources of variability - especially to explain the positioning of the fluid. .
Indeed, in Fig. 6, the authors write that " diversity in location of the ES can explain the experimental result with the co-existence of the T-reverse and T-shaped arrangements in similar AR " This was the part of the work I was less sure about. Isn't there to some degree a circular argument going on here? When the authors talk about the relative amount of fluid, and how it changes the packing, this is a highly non-trivial prediction, because it's not clear a priori how the two relate to each other… However, when looking at T-shapes or reverse T-shapes from the first figures, it's already clear that the fluid is located in different regions… but isn't that just a natural - and in fact inevitable consequence of the packing? It seemed to me, at least at first sight, that actively positioning the ES region in the simulation was close to inputting the results that the authors look for…In fact the manuscript finishes by the observation that "The results showed that the T-reversed case has more ES in the anterior side, whereas the T-shaped case has more ES in the posterior side", but as i mentioned this is something that is quite intuitive/visual from Fig. 1A. . . .
The authors mention in Materials and Methods that "As there are no data for the attraction between ABp cells and P2 cells (γ34), we used a similar scale for the attraction between the EMS cell and P2 cell (γ24)." … but isn't for instance variability on this parameter a strong candidate to explain the variability in the packings and the positioning of the fluid? This would seem at least superficially as a more direct causal mechanism?

On the effect of stochasticity, we agree with the reviewer that stochasticity in cell process can cause variability in cell arrangement even with the same AR. Actually, in our previous study, we have shown this point both experimentally and theoretically (Yamamoto & Kimura, Development, 2017). During the course of the revision, we decided not to argue "The coexistence of different types of cell arrangement within the same AR", but focus on the effect of local contour and the amount of ES. Therefore, we think we do not need to mention on the stochasticity in this new manuscript.

On the problem of cause vs effect on the location of fluid (ES), we decided to remove the argument of the location of fluid (ES). We understand the reviewer's concern that the location of ES can be affected by the cell arrangement, and we cannot specify which is the cause and effect. In the

© 2022. Published by The Company of Biologists under the terms of the Creative Commons Attribution License (https://creativecommons.org/licenses/by/4.0/).

model, we can control the initial location (as the cause), and it clearly affected the resultant cell arrangement (as shown in our previous manuscript). In contrast, for the real embryo, it is difficult to clarify the causal relationship without an experiment to manipulate the location of ES. We decided to leave this issue for our future study, and focus on the local contour and the amount of ES in this study. See also our response to Comment #2-1.

On the uncertainty on the attraction of ABp cell and P2 cell, it was misleading that we described “As there are no data for the attraction between ABp cells and P2 cells (γ34)”. While we could not observe the adhesion between ABp and P2 cells upon eggshell removal, we have experimental results to support our argument that γ34 is similar to γ24. The amount of cadherin molecules, which is responsible for the cell adhesion, is similar between ABp-P2 border and EMS-P2 border. In addition, the curvature of the cell near the cell adhesion, which is the indication of the strength of adhesion, is similar between ABp-P2 and EMS-P2 borders (Yamamoto & Kimura, Development, 2017). Therefore, it is unlikely that the uncertainty in this parameter is the cause of variability. We clarified this point in our revised manuscript (P. 23, L. 409–412).

Comment #3-2
Fig. 4 on the correlation between the type of packing and ES fraction:
I see the effect that the authors talk about, but given the amount of noise, is it significant? Furthermore, could the authors plot this in a slightly more “raw” manner, as looking at categories/bins can be a bit difficult to read… For instance, could they make a plot exactly like 4C, but where each point is color-coded in red or black if it’s a T-shape or a diamond? This would enable the readers to really see the full raw data and assess the dispersion.

Instead of the argument on the correlation, in the revised manuscript, we added new experimental data to demonstrate that the amount of ES (ES faction) can induce change in cell arrangement (Fig. 5 and Result Section 2.6). We believe the addition of this experiment clarify the reviewer’s concern.

Comment #3-3
Clarity of the presentation:
In general, the paper is quite hard to read and follow, because it goes back and forth between different types of theoretical investigates and data, but also back and forth between previous and current work from the authors.
In particular, the authors don’t explain enough the AA model (past paper), and its key differences with the current one. They keep referring to diverse results of their 2017 paper on this throughout the text, but this becomes a bit unclear. I think the authors should try to make this paper more self-contained. In particular, around page 7-8, they say that both models give different predictions on the T-reverse arrangement, and directly associate with the detailed shape of the egg… but given that the two models are quite different, isn’t there a lot other possibilities at this stage? Investigating the AA model with the right shape for instance would seem like the right control?

In the present manuscript, we reorganized the manuscript, so that we introduce experimental results at first (Fig. 1AB) and focus on the theoretical parts in the middle (Fig. 1C–4) before introducing new experimental results in Fig. 5. Afterwards, we go back to the theory in Fig. 6, while we hope this is acceptable.
Concerned to the difference between the current ‘Cell Morphology’ model and the previous ‘Anisotropic Attraction’ model, we revised the manuscript to explain both models well. Please see our response to Comment 2-5.
The assumptions for both models are basically same, while the description way is different mathematically. The reason why the Cell Morphology model reproduced the T-reverse shape is that this model can incorporate the accurate shape of the real embryo, as we emphasized in the manuscript. Incorporating the real shape in our previous model, which is suggested by the reviewer, is difficult. This is the reason why the Cell Morphology model is advantageous. Meanwhile, we have conducted control analyses from another direction. We had conducted Cell Morphology simulations with ellipsoidal shape and confirmed that they showed similar results as the previous model (Fig. 3 & 4). The results support the similarity in the two model.
Our other control analyses narrowed down that the precise shape of the embryo is the cause of the success of the Cell Morphology model to reproduce the T-reverse arrangement (Fig. 2 & 3).

© 2022. Published by The Company of Biologists under the terms of the Creative Commons Attribution License (https://creativecommons.org/licenses/by/4.0/).

Comment #3-4
The theory also seems quite dispersed in different figures, while compacting the experimental observations first might be better. For instance, Fig. 3 doesn't seem very necessary as the key information/model it contains seems to have been looked at much more clearly and systematically in Figure 5? (but are separated by a Fig. 4 of data)

We reorganized the figures for the theory part so that each figure delivers a clear message. In the present manuscript, each figure corresponds to each section of the results.
For the original Fig. 3 (Fig. 6 in the present manuscript), we remained the panels as it was. While we understand some redundancy between this figure and the original Fig. 5 (which is new Fig. 4), this figure argues that the surface tension has similar effect on the cell arrangement as the amount of ES. To emphasize this point, we thought we should compare in the same figure. We hope this is acceptable, and the overall organization of the manuscript was improved.

Comment #3-5
Minor comments:
Recent references such as Kuang et al, BioRxiv or Giammona et al, Plos Comp Biol 2021 also investigate this question of C elegans packing and seem relevant here.
Typos:
line 109: "regenerated actual eggshell shapes "—> i'm not sure what regenerated means in this context
line 186: "can cause variety "—> "can cause variability?/variations? "

We referred to the suggested papers (P.2, L.12; P.10, L.149; P.21, L.341). Thank you for the suggestions.
Concerned to the term "regeneration", we no longer use this expression in the present manuscript. We "extracted" actual eggshell shapes, and "reproduced" the cell arrangements.
Concerned to the term "variety", we revised to "variability". Thank you for pointing out.

Resubmission

First decision letter

MS ID#: DEVELOP/2021/200401

MS TITLE: The extra-embryonic space and the local contour are critical geometric constraints regulating cell arrangement

AUTHORS: Sungrim Seirin-Lee, Kazunori Yamamoto, and Akatsuki Kimura

I apologize I was unable to come back to you earlier. I have now received all the referees reports on the above manuscript, and have reached a decision. The referees' comments are appended below, or you can access them online: please go to BenchPress and click on the 'Manuscripts with Decisions' queue in the Author Area.

The overall evaluation is positive and we would like to publish a revised manuscript in Development, provided that the referees' comments can be satisfactorily addressed. Please attend to all of the reviewers' comments in your revised manuscript and detail them in your point-by-point response. If you do not agree with any of their criticisms or suggestions explain clearly why this is so.

We are aware that you may currently be unable to access the lab to undertake experimental revisions. If it would be helpful, we encourage you to contact us to discuss your revision in greater detail. Please send us a point-by-point response indicating where you are able to address concerns raised (either experimentally or by changes to the text) and where you will not be able to do so within the normal timeframe of a revision. We will then provide further guidance. Please also note that we are happy to extend revision timeframes as necessary.

© 2022. Published by The Company of Biologists under the terms of the Creative Commons Attribution License (https://creativecommons.org/licenses/by/4.0/).

Reviewer 1

Advance summary and potential significance to field

This revision sought to address concerns of the reviewers.
Comments for the author

I appreciate the points that the authors made that although Edgar et al. in 1994 showed the effect of removing the eggshell, this manuscript examines more precise roles for eggshell geometry and available space. However, I am not convinced that the conclusions reached significantly advance our understanding of development to a degree that it meets Development's criterion to "pose and test a significant hypothesis or address a significant question" and "provide novel perspectives that advance our understanding of development".

Reviewer 2

Advance summary and potential significance to field

Serin lee et al.
In this manuscript, Serin lee , Yamamoto and Kimura introduce a new phase-field model to predict blastomere arrangements at the 4 cell stage of C. elegans embryos and in other nematode species. This work improves upon a previous model by some of the same authors published in 2017, but remains timely in general given that the contribution of egg enveloping layers, or more generally of external constrains to cell positioning and thus embryo/tissue architecture remain underappreciated. The key novelty in the model is to describe the cell and egg shell boundaries using phase-field functions, which allows to bypass simplistic geometrical descriptions of cells/egg layers as ellipses, cubes or spheres. As demonstrated in the paper, this new model allows to predict arrangement, such as reverse T-shaped which previous models failed to predict presumably because these configurations become stable only under specific detailed geometrical parameters. In the MS, the model is systematically compared to experimental observations of blastomere arrangements in WT, mutants with abnormal egg shell shapes (higher aspect ratio), defects in cell-cell adhesion cell size, and even in multiple nematode species. A chief finding is that all observed blastomere arrangements may be well captured by two control parameters:
the aspect ratio of the egg shell and the extra embryonic space fraction (ES) which is essentially the volume in the egg shell divided by the sum of cell volumes. Interestingly ES provides an integrated measure of surface tension effects, cell-cell adhesion but also cell sizes compared to confinement size and thus may be considered as a better generic predictor for blastomere arrangements. My take is that this work is done with great care and control with the introduction of the phase-field method being completely new (at least to me). The output is somewhat qualitative, but of relevance to a general audience in development and morphogenesis typical of Development. I have few points which require clarification, but I am mostly supportive of publication.

Comments for the author

Detailed comments:
1- One aspect which was not clear is how ES is calculated from the images.
Inspection of DIC images for example in Fig 2A or 3A suggests that blastomere are completely plastered onto the egg shell. Yet the model below depicts a blue ES space between cells and the egg shell. Can the authors clarify this aspect and/or explain if this affects the predicted configuration?
2- A related point, is that to my understanding the ES factor is a complicated output of cell size, cell-cell adhesion and cortical tension. Yet this is not clearly formulated in the manuscript, and it reads as if the ES number is an independent parameter. If relevant, could the authors provide an analysis for a fixed egg shell geometry of how ES will depend on these biologically controlled parameters, so the reader can at least get a sense of this? For instance, in Figure 4A, the change of ES from 19% to 36% is achieved by simply shrinking cells, right? Similarly osmotic shocks experiments presumably affect cell sizes (and certainly tension as well). The authors should explain this better.

© 2022. Published by The Company of Biologists under the terms of the Creative Commons Attribution License (https://creativecommons.org/licenses/by/4.0/).

3- If I understood correctly, in the models, the egg shell is a fixed un-deformable boundary. How would the model prediction change if one considers a softer, more deformable elastic confining layer? This may be relevant to many embryos and tissues.
4- Cell positions. It was not obvious why in T-shaped and linear arrangements, the cells are always ordered in the same manner: Say, P2 is always at the posterior pole in linear embryos, and ABp always on top of EMS, in T-
shaped ones. Is this another important output of the model? Is it related to the history/timing or asymmetry of divisions? In any case, this should be explained/described, and maybe put on an equal foot as the predicted configurations.
5- The model as described does not provide any weight/probability associated to each configurations. This begs the question of how robust are the predictions to detailed parameters, especially geometry. Could the authors comment/document this aspect? Also are there hysteretic effects? For example starting from one 4-cell stage configuration, if one modulates the egg shell AR or ES% does the arrangement switches to the same configuration as that predicted from the histories of cell divisions?
6- There are few grammatical typos/difficult English formulations, which should be corrected: line 83 “value will go” etc…

Reviewer 3

Advance summary and potential significance to field

The authors propose a model to explain cell arrangement in C. elegans embryos at the 4-cells stage.
The previous model of the authors included cell-cell and cell-eggshell repulsion and attraction interactions as well as the aspect ratio of the eggshell modelled as an ellipsoid. Among the 5 configurations found in vivo (in WT and mutants), the previous model could recover only 4 configurations (Pyramid, Diamond, T-shaped and Linear), and missed the T-reverse arrangement that is found for embryos with increased AR and impaired adhesion. This is the author’s initial motivation for developing a new modeling approach.

In the new model presented in this paper, they switch from a model based on interacting soft spheres to a 2D multiphase-field model, which enables them to model precisely the eggshell contour, which is capsule-like in real-world embryos rather than ellipsoidal, and vary the amount of extra-embryonic space (ES).
These parameters, contour-shape and ES amount are thus allowed to vary on top of the other parameters either geometrical like the aspect ratio (AR), or physical like attraction/repulsion interactions and surface tension. The orientation of the division axis remains the same in all simulations and is derived from microscopy images.

In this new model, all the arrangements found in vivo can be recovered. In particular, the T-reverse arrangement is found, in agreement with experiments, for increased AR and decreased adhesion. This arrangement can be found in the new model thanks to incorporating the real local contour of the eggshell.

Next the authors turned to effect of ES amount on the arrangement according to their simulations. They varied the ES amount from 0 to 50 % for 3 different AR (1,8/ 2,2/ 2,6), either with ellipsoid shape or with real-word capsular shape, with or without adhesion. They underline that with all other parameters constant, the change in ES amount changes the cells arrangement. The sensitivity to ES amount change however, is dependant on the AR values. It is null for AR=1,8 (diamond for all parameters combinations), maximal for AR=2,2 and mild for AR=2,6. Furthermore, there is a particular range of ES amount, 19-22 % in which the presence or absence of adhesion makes a difference for cells arrangement.

The influence of ES amount on the cell arrangement was tested experimentally by increasing shell permeability and incubating in hypertonic KCl solution. The experiment was performed for 62 embryos, of different AR values and ES amount. The major features fitted with the simulation.

© 2022. Published by The Company of Biologists under the terms of the Creative Commons Attribution License (https://creativecommons.org/licenses/by/4.0/).

The authors then show, based on their simulation results, that increasing or decreasing the ES amount has the same effect as increasing or decreasing the surface tension. Indeed, departing from a shell which shape would give the normal diamond arrangement (EggD, AR=1,8, real-world contour shape, no adhesion), increasing the surface tension, or increasing the ES amount both lead to a T-reverse arrangement instead of diamond. Conversely, departing from a shell which shape would give the T-reverse arrangement (Egg Tr, AR=2,2, real-world contour shape, no adhesion), decreasing the surface tension, or decreasing the ES amount both lead to diamond shape.
Last, the authors propose that the variation in ES amount can be the cause of diversity of cell arrangements across different nematodes species. They compared 5 nematode species, retrieving the AR and ES amount. In agreement with their simulation, when the AR is <=1,8, the diamond shape is predominant. For AR>1,8, the non-diamond arrangements are found, depending on the ES amount.

The authors enhance the interest of using multi-phase field model in describing the precise shape of eggshells, and justify the use of 2D model as sufficient to describe the arrangements addressed in this paper, as checked on test simulations, although the 3D model can be implemented with the same formulation but at higher computationnal costs. They emphasize the benefits of this model compared to the previous one, as the phase-field model enables to incorporate the eggshell contrary to the previous formulation. The authors underline that even though they focused on the influence of the geometric features in this study, their model is also suitable to study the influence of the physical features
(interactions, orientation of division) and their interplay.

Comments for the author

1. The main issue that needs to be addressed is the how the phase field model is introduced. Equation 1 is given as being the general form of the phase field modeling but with little explanation. As effective as it can be to model the shape of the embryo in the following, this model is not introduce properly for the audience of the journal Development. Indeed, one would expect that the intuition behind the equation is explained as well as a description of where this type of modelling have been applied before. Moreover the theoretical justification of why such a model would be applicable to a system like a developing embryo should appear in the introduction or the part 2.1.
2. The author claim that it is accurate enough to use the 2D version of the model. Since the 3D and 2D models are only tested in one configuration and compared qualitatively, it is unclear how generalisable to 3D the results are. This is a crucial point too, since the arguments that are made in 2D might not hold in 3D.
3. the question of the orientation of cell division should be discussed earlier in the paper, even though the solution was that it is always the same plane of division.

Minor remarks:
1)Figure 4, and in general in the section 2.5 (results about the effect of ES on cell arrangement) : why is T arrangement (not reverse) not found in any of the condition ? Is it because it is transient, before becoming linear ? Experimentally (section 2.6), T-shaped does exist among the 62 embryos sampled.
2)In section 2.6 (experimental evidence that ES amount affects celle rearrangements) : the different AR are from natural variability ?
3)In section 2.7, figure 6 (about change in ES having same effect as the change in surface tension) For EggD, how much was the ES amount increased ? More than 50 %? Because if not, how comes they don’t show this arrangement in Figure 4, for the combination Egg-C, AR=1,8 , no adhesion ? In Figure there is only diamond shape for all ES amount up to 50 %.
4) On Figure 7, I suggest to put the markers (cross , square, etc) below each name of species on x-axis of Figure7A.
5) please rewrite the sentence starting on line 104 " We name ... " for clarity 6) line 203, I think you mean "combined" instead of "combinatorial"
7) line 227, "significant concentration is unclear"
8) line 620, rephrase the sentence that starts with "For the technical .." what do you mean? All the necessary information should be in the article.

© 2022. Published by The Company of Biologists under the terms of the Creative Commons Attribution License (https://creativecommons.org/licenses/by/4.0/).

First revision

Author response to reviewers' comments

Point-by-point responses to comments made on the previous version of the manuscript (The original comments are italicised.)

In the revised manuscript, changes are highlighted in blue texts.

Reviewer 1 Advance Summary and Potential Significance to Field:
This revision sought to address concerns of the reviewers.

Reviewer 1 Comments for the Author:
I appreciate the points that the authors made that although Edgar et al. in 1994 showed the effect of removing the eggshell, this manuscript examines more precise roles for eggshell geometry and available space. However, I am not convinced that the conclusions reached significantly advance our understanding of development to a degree that it meets Development's criterion to "pose and test a significant hypothesis or address a significant question" and "provide novel perspectives that advance our understanding of development".

Our Response: We appreciate the reviewer to notice that our study is novel as it revealed the precise roles for eggshell geometry and available space. Unfortunately (for us), this reviewer evaluated the novelty is not large enough to reach the standard of *Development*. We believe our finding is novel enough to be published in *Development*. We posed and tested a hypothesis that slight change in the shape and amount of available space affected cell arrangements during development. We provided novel perspectives that the available space is an important factor to control cell arrangement.

Reviewer 2 Advance Summary and Potential Significance to Field:
Serin lee et al.
In this manuscript, Serin lee , Yamamoto and Kimura introduce a new phase-field model to predict blastomere arrangements at the 4 cell stage of C. elegans embryos and in other nematode species. This work improves upon a previous model by some of the same authors published in 2017, but remains timely in general, given that the contribution of egg enveloping layers, or more generally of external constrains to cell positioning and thus embryo/tissue architecture remain underappreciated. The key novelty in the model is to describe the cell and egg shell boundaries using phase-field functions, which allows to bypass simplistic geometrical descriptions of cells/egg layers as ellipses, cubes or spheres. As demonstrated in the paper, this new model allows to predict arrangement, such as reverse T-shaped which previous models failed to predict, presumably because these configurations become stable only under specific detailed geometrical parameters. In the MS, the model is systematically compared to experimental observations of blastomere arrangements in WT, mutants with abnormal egg shell shapes (higher aspect ratio), defects in cell-cell adhesion, cell size, and even in multiple nematode species. A chief finding is that all observed blastomere arrangements may be well captured by two control parameters: the aspect ratio of the egg shell and the extra embryonic space fraction (ES) which is essentially the volume in the egg shell divided by the sum of cell volumes. Interestingly ES provides an integrated measure of surface tension effects, cell-cell adhesion but also cell sizes compared to confinement size, and thus may be considered as a better generic predictor for blastomere arrangements. My take is that this work is done with great care and control, with the introduction of the phase-field method being completely new (at least to me). The output is somewhat qualitative, but of relevance to a general audience in development and morphogenesis typical of Development. I have few points which require clarification, but I am mostly supportive of publication.

Reviewer 2 Comments for the Author:

Detailed comments:
1-One aspect which was not clear is how ES is calculated from the images. Inspection of DIC images for example in Fig 2A or 3A suggests that blastomere are completely plastered onto the

© 2022. Published by The Company of Biologists under the terms of the Creative Commons Attribution License (https://creativecommons.org/licenses/by/4.0/).

egg shell. Yet the model below depicts a blue ES space between cells and the egg shell. Can the authors clarify this aspect, and/or explain if this affects the predicted configuration?

Our Response:
We thank reviewer's careful reading and indications. From the microscope images, we calculated ES through a straightforward tracing of both the blastomere cells and the eggshell. We understand the reviewer's concern that, in the simulation images (e.g. Fig. 2), a blue region along the eggshell looks as if it is a part of ES (and the border of the white and blue region looks as if it is the eggshell). However, this is not the case. The blue region is the eggshell itself (it is thicker than the real eggshell for a calculation purpose). The actual ES is further inside the region, which is colored with darker blue. In other words, when we calculated ES, we excluded the thickness of the eggshell. In order to clarify the definition of the ES, we added a description in main text (p. 11) with further informative figure in Fig.1C to avoid the confusion. We also provided a detailed mathematical definition of ES in Materials and Method 4.3, p.26.

2-A related point, is that to my understanding the ES factor is a complicated output of cell size, cell-cell adhesion and cortical tension. Yet this is not clearly formulated in the manuscript, and it reads as if the ES number is an independent parameter. If relevant, could the authors provide an analysis for a fixed egg shell geometry of how ES will depend on these biologically controlled parameters, so the reader can at least get a sense of this? For instance, in Figure 4A, the change of ES from 19% to 36% is achieved by simply shrinking cells, right? Similarly osmotic shocks experiments, presumably affect cell sizes (and certainly tension as well). The authors should explain this better.

Our Response:
The reviewer is correct that ES is the output of multiple factors both in real embryo and in our model. Therefore, the ES is calculated from the output images not only for the experiments but also for the modeling. We added more detailed explanation in main text (p.11) with ES definition as we answered in the previous comment.

In Fig. 4, we changed ES ratio by simply changing the target volume parameters (*Vm* in Eq. 1). In Fig. 4, the other model parameters were fixed. In contrast, as the reviewer pointed out, the *Vm* parameter is not the sole determinant of the cell size, and thus ES. The cell size and ES changes by changing other parameters, as we demonstrated for the surface tension in Fig. 6A and B. We provided the number of ES in Fig. 6.

For the experiment part, we agree that the change in osmolarity may not affect only the cell size, but also the surface tension. Therefore, we cannot exclude the possibility that the change in the cell arrangement in the experiment of Fig. 5 is induced by other factors than cell size. A good news for us is that our model indicates that the direct control in cell size and surface tension induce interchangeable effect on the resultant cell size and ES (Fig. 6), as discussed in the earlier part of this comment. Therefore, the experimental correlation between the ES and the cell arrangement (Fig. 5) is consistent with our model, even if the change in ES is induced by the change in surface tension. We added this discussion in the revised manuscript (p.16, 271–277).

3- If I understood correctly, in the models, the egg shell is a fixed un-deformable boundary. How would the model prediction change if one considers a softer, more deformable elastic confining layer? This may be relevant to many embryos and tissues.

Our Response:
Thank you for very interesting question. In this study, we fixed the eggshell because the eggshell of the nematodes' embryo is very hard and does not change the shape. However, our mathematical model using phase-field method can be easily applied for the case of deformable eggshell. Deformable domain case has been already tried with the phase-field modeling in our previous work, where we applied the method to chromatin dynamics (S. Seirin-Lee et al., Role of dynamic nuclear deformation on genomic architecture reorganization. PLOS Computational Biology (2019) 15 (8): e1007289 DOI:10.1371/journal.pcbi.1007289). For cellular systems, our model can be simply extended to the deformable eggshell case and deformable boundary of tissues as well. We added this point in the revised manuscript (P.22, L.391-P.23, 394).

We decided not to perform a modeling with soft eggshell in the present study, because such situation is not observed for the nematodes' eggshell (to our knowledge) and the prediction has little biological implication. As a future work, we would like to search for a suitable

© 2022. Published by The Company of Biologists under the terms of the Creative Commons Attribution License (https://creativecommons.org/licenses/by/4.0/).

biological system to apply our method to analyze cell arrangements in deformable boundaries.

4- Cell positions. It was not obvious why in T-shaped and linear arrangements, the cells are always ordered in the same manner: Say, P2 is always at the posterior pole in linear embryos, and ABp always on top of EMS, in T-shaped ones. Is this another important output of the model? Is it related to the history/timing or asymmetry of divisions? In any case, this should be explained/described, and maybe put on an equal foot as the predicted configurations.

Our Response:
We added the explanation in the introduction (P.3, L.32–34). We don't think it is an important output of the model, but rather an obvious one based on the definition of the cells. P2 is the posterior daughter of the P1 cell, which is the posterior daughter of the one-cell, P0. Therefore, it is reasonable that the P2 is always at the posterior pole in linear embryos. Similarly, for the case of ABp-EMS attachment, ABp is defined as the posterior daughter of AB cell. Therefore, ABp attaching more to EMS than ABa is not surprising.

5- The model as described does not provide any weight/probability associated to each configurations. This begs the question of how robust are the predictions to detailed parameters, especially geometry. Could the authors comment/document this aspect? Also are there hysteretic effects? For example starting from one 4-cell stage configuration, if one modulates the egg shell AR or ES% does the arrangement switches to the same configuration as that predicted from the histories of cell divisions?

Our Response:
This is a very interesting point. The present model is a deterministic model and the outcome of the model is determined by the initial condition. The robustness of the predictions is evaluated by changing/fluctuating the initial conditions. For the cell sizes, adhesions and surface tensions, we tested various values and found robustness/sensitivity of the outcomes for these parameters. In contrast, we did not change values for cell division timing, cell division axis or other conditions in the present study. These factors might be important for hysteresis effect. However, examining the sensitivity against all factors in this paper in beyond the scope of the manuscript, and we concluded that it makes the main focus of this study unclear. Please let us save these topics for our future studies.

We would like to point out that our previous model included fluctuations in cell division axis and cell movement (Yamamoto & Kimura, Development, 2017), and made probabilistic predictions. This made the transition from one configuration to another dependent on the parameter values gradual, but there were no apparent biological implications related to the stochasticity. We would like to also point out that, in the previous study, we have examined the contribution of cell division axis and timing in the model.

We have commented this point in Discussion (P.23, L.397) and would like to remine the details for the future work.

6- There are few grammatical typos/difficult English formulations, which should be corrected: line 83 "value will go" etc…

Our Response: We have asked a professional English editing service to check the English of the manuscript. We believe this concern is solved in the revised manuscript.

Reviewer 3 Advance Summary and Potential Significance to Field:
The authors propose a model to explain cell arrangement in C. elegans embryos at the 4-cells stage. The previous model of the authors included cell-cell and cell-eggshell repulsion and attraction interactions as well as the aspect ratio of the eggshell modelled as an ellipsoid. Among the 5 configurations found in vivo (in WT and mutants), the previous model could recover only 4 configurations (Pyramid, Diamond, T-shaped and Linear), and missed the T-reverse arrangement that is found for embryos with increased AR and impaired adhesion. This is the author's initial motivation for developing a new modeling approach.

In the new model presented in this paper, they switch from a model based on interacting soft spheres to a 2D multiphase-field model, which enables them to model precisely the eggshell contour, which is capsule-like in real-world embryos rather than ellipsoidal, and vary the amount of

© 2022. Published by The Company of Biologists under the terms of the Creative Commons Attribution License (https://creativecommons.org/licenses/by/4.0/).

extra-embryonic space (ES). These parameters, contour-shape and ES amount are thus allowed to vary on top of the other parameters, either geometrical like the aspect ratio (AR), or physical like attraction/repulsion interactions and surface tension. The orientation of the division axis remains the same in all simulations and is derived from microscopy images.

In this new model, all the arrangements found in vivo can be recovered. In particular, the T-reverse arrangement is found, in agreement with experiments, for increased AR and decreased adhesion. This arrangement can be found in the new model thanks to incorporating the real local contour of the eggshell.

Next the authors turned to effect of ES amount on the arrangement according to their simulations. They varied the ES amount from 0 to 50 % for 3 different AR (1,8/ 2,2/ 2,6), either with ellipsoid shape or with real-word capsular shape, with or without adhesion. They underline that with all other parameters constant, the change in ES amount changes the cells arrangement. The sensitivity to ES amount change, however, is dependant on the AR values. It is null for AR=1,8 (diamond for all parameters combinations), maximal for AR=2,2 and mild for AR=2,6. Furthermore, there is a particular range of ES amount, 19-22 % in which the presence or absence of adhesion makes a difference for cells arrangement.

The influence of ES amount on the cell arrangement was tested experimentally by increasing shell permeability and incubating in hypertonic KCl solution. The experiment was performed for 62 embryos, of different AR values and ES amount. The major features fitted with the simulation.

The authors then show, based on their simulation results, that increasing or decreasing the ES amount has the same effect as increasing or decreasing the surface tension. Indeed, departing from a shell which shape would give the normal diamond arrangement (EggD, AR=1,8, real-world contour shape, no adhesion), increasing the surface tension, or increasing the ES amount both lead to a T-reverse arrangement instead of diamond. Conversely, departing from a shell which shape would give the T-reverse arrangement (Egg Tr, AR=2,2, real-world contour shape, no adhesion), decreasing the surface tension, or decreasing the ES amount both lead to diamond shape.

Last, the authors propose that the variation in ES amount can be the cause of diversity of cell arrangements across different nematodes species. They compared 5 nematode species, retrieving the AR and ES amount. In agreement with their simulation, when the AR is <=1,8, the diamond shape is predominant. For AR>1,8, the non-diamond arrangements are found, depending on the ES amount.

The authors enhance the interest of using multi-phase field model in describing the precise shape of eggshells, and justify the use of 2D model as sufficient to describe the arrangements addressed in this paper, as checked on test simulations, although the 3D model can be implemented with the same formulation but at higher computationnal costs. They emphasize the benefits of this model compared to the previous one, as the phase-field model enables to incorporate the eggshell contrary to the previous formulation. The authors underline that even though they focused on the influence of the geometric features in this study, their model is also suitable to study the influence of the physical features (interactions, orientation of division) and their interplay.

Reviewer 3 Comments for the Author:

1. The main issue that needs to be addressed is the how the phase field model is introduced. Equation 1 is given as being the general form of the phase field modeling but with little explanation. As effective as it can be to model the shape of the embryo in the following, this model is not introduce properly for the audience of the journal Development. Indeed, one would expect that the intuition behind the equation is explained as well as a description of where this type of modelling have been applied before. Moreover the theoretical justification of why such a model would be applicable to a system like a developing embryo should appear in the introduction or the part 2.1.

Our Response: Thank you for the referee's careful reading. We revised the explanation about phase-field model in the main text from biologists' point of view so that the readers of *Development* will understand the essence of the method (P.4, L.70-P.6, L.88). We hope the revised explanation satisfactory address the reviewer's concern.

© 2022. Published by The Company of Biologists under the terms of the Creative Commons Attribution License (https://creativecommons.org/licenses/by/4.0/).

We suspect that the difficulty in understanding the formulation of the phase-field modeling method comes from the fact that it is fundamentally different format from a common biological model based on differential equations. The phase-field modeling is formulated by functional energy forms. For the readers interested in this aspect, we provided a detailed formation of energy functionals in Supplementary Information (P.1).

2. The author claim that it is accurate enough to use the 2D version of the model. Since the 3D and 2D models are only tested in one configuration and compared qualitatively, it is unclear how generalisable to 3D the results are. This is a crucial point too, since the arguments that are made in 2D might not hold in 3D.

Our Response: First of all, we would like to point out that we presented the consistency between the 2D and 3D models in the paper by showing four configurations (not only in Fig. 1F but also 3 configurations in Fig. S2), resulting in three distinct cell arrangements. Further, we tested 3D model with more than 50 different parameter sets and did not find notable differences to affect the conclusion of our results. We hope these multiple investigations are enough.

In case the reviewer still questions the reliability of the 2D model, we would like to add reasons why 2D model is enough in the case of this particular study, as follows. Because the geometric centers (e.g. the cell nuclei) of the 4 cells and the eggshell is on the 2D plane, the major forces controlling the location of the cell should be generated on the plane, and act along the plane. If this is not the case, the centers of the cells will be out of the plane, which is not the case in the real embryos. Therefore, as far as the geometric centers of the cells are moving on a 2D plane, the calculation in 2D space should be enough (P.6, L.104–P.7, L.108). Our demonstration of the 3D simulation of four different configurations resulting in three distinct cell arrangements is supporting this idea (Figs. 1F and S2). In conclusion, we cannot think of any logical reason to expect fundamental difference between 2D and 3D to challenge the conclusion of this study. Interestingly, 2D and 3D models is consistent with each other not only in a qualitative manner, but also in a quantitative manner. ES ratio that changes the arrangement in 3D is similar scale with 2D case (See Fig.S2 and Fig.4B (Egg-C)).

3. the question of the orientation of cell division should be discussed earlier in the paper, even though the solution was that it is always the same plane of division.

Our Response: We have revised the manuscript as requested by the reviewer (P.6, L.104–P7,107).

Minor remarks:
1) Figure 4, and in general in the section 2.5 (results about the effect of ES on cell arrangement) : why is T arrangement (not reverse) not found in any of the condition ? Is it because it is transient, before becoming linear ? Experimentally (section 2.6), T-shaped does exist among the 62 embryos sampled.

Our Response: Yes, it is because T-shape is observed transiently in our model (P.7, L.116–120). Fig. 4 describes the cell arrangement at the equilibrium state. It should be noted that the timing of observing T-shape in the model is consistent with the timing of cell division. Therefore, the model accounts also for the T-shape arrangement.

2) In section 2.6 (experimental evidence that ES amount affects celle rearrangements) : the different AR are from natural variability ?

Our Response: It is not from natural variability. We manipulated the AR using *lon-1* mutation and the knockdown of *C27D9.1* gene. These manipulations were used in our previous study examining the effect of changing AR (Yamamoto & Kimura, *Development* 2017). The manipulation is clarified in the legend of Fig. 5 and the Methods (P.28, L.515- 522).

3) In section 2.7, figure 6 (about change in ES having same effect as the change in surface tension) For EggD, how much was the ES amount increased ? More than 50 %? Because if not, how comes they don't show this arrangement in Figure 4, for the combination Egg-C, AR=1,8 , no adhesion ? In Figure, there is only diamond shape for all ES amount up to 50 %.

© 2022. Published by The Company of Biologists under the terms of the Creative Commons Attribution License (https://creativecommons.org/licenses/by/4.0/).

Our Response: Thank you for pointing out. This part was confusing and misleading in the previous version.

The condition for Fig. 6C (T-reverse) was Egg-D shape, ES=36% AR=1.8 and no adhesion (In the revised paper, we replaced Fig. 6C with the data for ES=31 % to make the comparison easier to understand). The difference between Fig. 4A (Diamond arrangement for ES=0-50%, AR=1.8, and no adhesion) was the precise contour. In more detail, we confirmed the effect of the precise contour between Egg-D and Egg-E/Egg-C (Supplementary Fig. S3, ES=32%) for the same ES ratios of ES=28%, 32 % cases (We only added 32% result in the paper). Therefore, this was another demonstration that the precise contour (Egg-E/C or Egg-D) affects the cell arrangement.
It was misleading that we concluded that diamond-type arrangement always appears for AR=1.8 regardless of the eggshell shape, only from the result of Egg-E and -C. We revised this part (P.13, L.206–213).

In contrast, and interestingly, the eggshell-shape-dependent transition in cell arrangement for AR=1.8 was detected only for the adhesion-absent case but not for the adhesion-present case (Supplementary Fig. S3, ES=32 %). This is new evidence that the adhesion acts to increase the robustness against the precise of eggshell shape, in addition to the robustness against the aspect ratio as we demonstrated before (Yamamoto & Kimura, *Development* 2017). We added this discussion in the revised manuscript (P.13, L.206-213).

4) On Figure 7, I suggest to put the markers (cross , square, etc) below each name of species on x-axis of Figure7A.

Our Response: As suggested, we added the markers in the x-axis of Fig.7A.

5) please rewrite the sentence starting on line 104 " We name ... " for clarity

Our Response: We revised the sentence as follows (P. 7, L.116–122):
"We named the eggshell shape extracted from a wild-type embryo as Egg-D. Egg-D has an AR of 1.8, and shows Diamond-type cell arrangement in the experiment. Eggshell shape from a lon-1 mutation was named Egg-Ts, which has the AR of 2.2 and showed T-shaped arrangement. Similarly, Egg-L is the eggshell shape of a C27D9.1 gene knockdown lon-1 mutant embryos, with AR=2.6 that showed Linear arrangement (Fig. 1E)."
We hope the sentence clarifies the meaning.

6) line 203, I think you mean "combined" instead of "combinatorial"

Our Response: We revised the word as suggested (P.14, L.224).

7) line 227, "significant concentration is unclear"

Our Response: We revised the sentence as follows (P.14, L.247): *"This calculation supported our argument from a statistical viewpoint."* We hope the revised sentence clarifies the meaning.

8) line 620, rephrase the sentence that starts with "For the technical .." what do you mean? All the necessary information should be in the article.

Our Response: This sentence meant that basic technic of phase-field modeling can be found the addressed reference. All the information is in this article for modeling. We revised the sentence for readers to be able to find a good reference about introduction of phase-field method (Supplementary information P.1).

© 2022. Published by The Company of Biologists under the terms of the Creative Commons Attribution License (https://creativecommons.org/licenses/by/4.0/).

Second decision letter

MS ID#: DEVELOP/2021/200401

MS TITLE: The extra-embryonic space and the local contour are critical geometric constraints regulating cell arrangement

AUTHORS: Sungrim Seirin-Lee, Kazunori Yamamoto, and Akatsuki Kimura

I have now received all the referees reports on the above manuscript, and have reached a decision. The referees' comments are appended below, or you can access them online: please go to BenchPress and click on the 'Manuscripts with Decisions' queue in the Author Area.

The overall evaluation is very positive and we would like to publish your manuscript in Development. Before we can proceed with acceptance please address the few comments from Reviewer 1.

Reviewer 2

Advance summary and potential significance to field

This work is an important advance both methodological and conceptual to understand cell positions during early embryo development.

Comments for the author

The authors have adressed all my questions in a satisfactory manner.

Reviewer 3

Advance summary and potential significance to field

The authors have answered my remarks and I'm overall supportive of publication. However, for the presentation of the model, I am not sure that the first equation is necessary to understand the story and it might deter equation-adverse readers to look further into the article.

The aim of this paper is not so much to justify the use of a multiphase-field model, which was my initial impression. Instead this model is used as a tool to inquire about the role of the geometric constraints of the early egg shell, in regulating cell arrangement. In that sense, this paper highlights a biological phenomenon that might usually be overlooked and is of interest for the developmental biology community. This would indeed add to current discussions about spatial arrangements of small cell clusters.

Comments for the author

suggestion to authors:
1)p.6: I'm questioning the interest of having equation (1) in the main if the only interpretation of it is that "In brief, φm changes over time to make the value of Am zero, which is the equilibrium state." It would probably be better to only keep equation (2) whose terms are discussed in the text. In the current presentation on p.6, the term g_adh(\phi_m, \phi_i) is misleading, because, as it is shown in the supplementary, it is actually a sum over i and not a term indexed by i. I suggest to refer to the equation that characterize g_adh in the supplementary p.24 . And it would be more clear if it was written as g_adh(¥phi _m, ¥phi), i.e. removing the index i.
2) p.4: this sentence is grammatically incorrect "we applied the model to input various shapes of the eggshell and successfully presented dynamic changes in cell arrangement as an output."
3) is the code available? This would be necessary to reproduce the results

© 2022. Published by The Company of Biologists under the terms of the Creative Commons Attribution License (https://creativecommons.org/licenses/by/4.0/).

Second revision

Author response to reviewers' comments

Point-by-point responses to comments made on the previous version of the manuscript (The original comments are italicised.)
In the revised manuscript, changes are highlighted in blue texts.

Reviewer 3
The authors have answered my remarks and I'm overall supportive of publication. However, for the presentation of the model, I am not sure that the first equation is necessary to understand the story and it might deter equation-adverse readers to look further into the article.
The aim of this paper is not so much to justify the use of a multiphase-field model, which was my initial impression. Instead, this model is used as a tool to inquire about the role of the geometric constraints of the early egg shell, in regulating cell arrangement. In that sense, this paper highlights a biological phenomenon that might usually be overlooked and is of interest for the developmental biology community. This would indeed add to current discussions about spatial arrangements of small cell clusters.

Reviewer 3 Comments for the Author:
suggestion to authors:

1) p.6: I'm questioning the interest of having equation (1) in the main if the only interpretation of it is that "In brief, φm changes over time to make the value of Am zero, which is the equilibrium state." It would probably be better to only keep equation (2) whose terms are discussed in the text.

Our Response: We understand the reviewer's concern. However, we decided not to remove the equation (1). The removal of eq. (1), while leaving only eq. (2), is not mathematically correct. The removal of the master equation (1) in the text will cause significant confusion for many readers in the fields of interdisciplinary research, biophysics, and mathematical biology. *Development* is one of the leading biological journals referenced by theoretical scientists. We believe that the readers will not give up reading our paper only by one mathematical equation, because the biological issues are very much emphasized in our manuscript. We constructed the manuscript carefully so that readers from various background can understand.

In the current presentation on p.6, the term g_adh(¥phi_m, ¥phi_i) is misleading, because, as it is shown in the supplementary, it is actually a sum over i and not a term indexed by i. I suggest to refer to the equation that characterize g_adh in the supplementary p.24 . And it would be more clear if it was written as g_adh(¥phi_m, ¥phi), i.e. removing the index i.

Our Response: Thank you very much for reviewer's careful reading. We revised the manuscript according to the reviewer's comment. The index "*i*" in *¥phi_i* had been used in duplicate in the summation symbol of definition *g_adh*. Because *g_adh(¥phi_m, ¥phi)* is not correct mathematically, we changed the index symbol "*i*" to "*j*" and also revised the main manuscript more carefully. Now, the descriptions became mathematically correct (p. 6 and p.24).

2) p.4: this sentence is grammatically incorrect "we applied the model to input various shapes of the eggshell and successfully presented dynamic changes in cell arrangement as an output."

Our Response: We revised the sentence "Using this to our advantage, we applied the model to input various shapes of the eggshell and successfully demonstrated the dynamic changes in cell arrangements as outputs." (p.4)

3) is the code available? This would be necessary to reproduce the results

Our Response: Yes. The codes are available on request, as we stated it in the text (P.30).

© 2022. Published by The Company of Biologists under the terms of the Creative Commons Attribution License (https://creativecommons.org/licenses/by/4.0/).

Third decision letter

MS ID#: DEVELOP/2021/200401

MS TITLE: The extra-embryonic space and the local contour are critical geometric constraints regulating cell arrangement

AUTHORS: Sungrim Seirin-Lee, Kazunori Yamamoto, and Akatsuki Kimura
ARTICLE TYPE: Research Article

I am happy to tell you that your manuscript has been accepted for publication in Development, pending our standard ethics checks.

© 2022. Published by The Company of Biologists under the terms of the Creative Commons Attribution License (https://creativecommons.org/licenses/by/4.0/).
